# Orthogonal representations for robust context-dependent task performance in brains and neural networks

**DOI:** 10.1016/j.neuron.2022.01.005

**Published:** 2022-04-06

**Authors:** Timo Flesch, Keno Juechems, Tsvetomira Dumbalska, Andrew Saxe, Christopher Summerfield

**Affiliations:** 1 Department of Experimental Psychology, University of Oxford, Oxford OX2 6GG, UK; 2 St. John’s College, University of Oxford, Oxford OX1 3JP, UK; 3 Gatsby Computational Neuroscience Unit & Sainsbury Wellcome Centre, University College London, London, UK; 4 CIFAR Azrieli Global Scholars program, CIFAR, Toronto, ON, Canada

**Keywords:** representational geometry, artificial neural networks, task learning, functional magnetic resonance imaging, orthogonal manifolds

## Abstract

How do neural populations code for multiple, potentially conflicting tasks? Here we used computational simulations involving neural networks to define “lazy” and “rich” coding solutions to this context-dependent decision-making problem, which trade off learning speed for robustness. During lazy learning the input dimensionality is expanded by random projections to the network hidden layer, whereas in rich learning hidden units acquire structured representations that privilege relevant over irrelevant features. For context-dependent decision-making, one rich solution is to project task representations onto low-dimensional and orthogonal manifolds. Using behavioral testing and neuroimaging in humans and analysis of neural signals from macaque prefrontal cortex, we report evidence for neural coding patterns in biological brains whose dimensionality and neural geometry are consistent with the rich learning regime.

## Highlights

• We trained artificial neural networks and humans on context-dependent decision tasks
• Initial weight variance determined the network’s representational geometry
• Human fronto-parietal representations were similar to those of low-variance networks
• Theory of nonlinear gating explains how these are formed in neural networks and brains

## Teaser

How do brains represent multiple tasks? The authors use artificial neural networks to derive a computational theory of neural coding and validate its predictions in recordings of human and macaque brains. The findings suggest that task-specific information is represented along orthogonal coding axes, which minimizes interference and maximizes robustness to noise.

## Introduction

Humans and other primates can exhibit versatile control over behavior in rapidly changing contexts (Passingham and Wise, 2012). For example, we can switch nimbly between sequential tasks that require distinct responses to the same input data, as when alternately judging fruit by shape or size and friends by gender or age (Roy et al., 2010; Mante et al., 2013; Saez et al., 2015; Takagi et al., 2020). Human studies have mapped the brain regions that exert control during task performance (Koechlin et al., 2003; Kerns et al., 2004; Yeung et al., 2006; Cole et al., 2016) or measured the processing costs incurred by task switches (Monsell, 2003; Brown et al., 2007). However, how the neural representations that support context-dependent task performance are acquired remains a key open question for cognitive and neural scientists (Gao and Ganguli, 2015; Mastrogiuseppe and Ostojic, 2018; Cueva et al., 2020; Dubreuil et al., 2020; Badre et al., 2021; Freund et al., 2021).

One recently popular theory proposes that stimulus and context signals are projected into a high-dimensional neural code, permitting linear decoding of exhaustive combinations of task variables (Fusi et al., 2016). Indeed many neurons, especially in prefrontal and parietal cortex, exhibit nonlinear mixed selectivity, multiplexing information over several potentially relevant task variables (Rigotti et al., 2013; Raposo et al., 2014; Tang et al., 2019) with errors heralded by a collapse in dimensionality (Rigotti et al., 2013). This high-dimensional random mixed selectivity offers great behavioral flexibility because it maximizes the potential for discrimination among diverse combinations of inputs but also implies that neural codes should be relatively unstructured and task agnostic. An alternative theory states that neural representations are mixed selective but structured on a low-dimensional and task-specific manifold (Ganguli et al., 2008; Sadtler et al., 2014; Gao and Ganguli, 2015; Chaudhuri et al., 2019; Cueva et al., 2020) where correlated patterns of firing confer robustness on the population code (Zohary et al., 1994). Representations may adapt so that irrelevant task information is wholly or partially filtered out in ways that minimize interference between tasks (Cohen et al., 1990; Miller and Cohen, 2001), consistent with accounts emphasizing that neural codes are sculpted by task demands (Duncan, 2001) or through attention to scenes and objects (Çukur et al., 2013). The question of whether neural codes are task agnostic or task specific speaks to core problems in neural theory with widespread implications for understanding the coding properties of neurons and neural populations (Yuste, 2015; Saxena and Cunningham, 2019).

Here we studied the dimensionality and geometry of neural codes supporting sequential context-dependent task performance in both neural networks and the human brain. We first formalized a continuum of solutions to the problem using the framework provided by feedforward neural networks. An emergent theme in machine learning research is that neural networks can solve nonlinear problems in two distinct ways, dubbed the “lazy” and “rich” regimes, which, respectively, give rise to high- and low-dimensional representational patterns in the network hidden units (Chizat et al., 2018; Jacot et al., 2018; Arora et al., 2019; Lee et al., 2019; Woodworth et al., 2020). In the lazy regime, which occurs when weights in the hidden layers are initialized with draws from a distribution with high variance, the dimensionality of the input signals is expanded via random projections to the hidden layer such that learning is mostly confined to the readout weights. In the rich regime, which occurs under low initial variance, the hidden units instead learn highly structured representations that are tailored to the task demands (Saxe et al., 2019; Geiger et al., 2020; Woodworth et al., 2020; Paccolat et al., 2021). We used neural network simulations to characterize the nature of these solutions for a canonical context-dependent decision-making setting and employed representational similarity analysis to explore their neural geometry. Subsequently, we compared these observations to BOLD (blood-oxygen-level-dependent) data recorded when humans performed an equivalent task and to neural signals previously recorded from macaque prefrontal cortex (PFC) during context-dependent decisions (Mante et al., 2013). In humans, we found that dorsal portions of the PFC and posterior parietal cortex share a neural geometry and dimensionality with networks that are trained in the rich regime. This solution involves representing distinct tasks as low-dimensional and task-specific neural manifolds in a way that minimizes interference and maximizes robustness among potentially competing responses (Koay et al., 2019). Task-relevant features were mapped onto orthogonal dimensions in neural state space. Relative to these, task-irrelevant features were strongly attenuated, suggestive of a rich encoding scheme. Neural signals in the two monkeys were heterogenous but we see strong support for these orthogonal manifolds in one animal, with neural signals in the other strongly biased toward a single input dimension as previously reported (Mante et al., 2013; Aoi et al., 2019).

## Results

**Figure 1:**
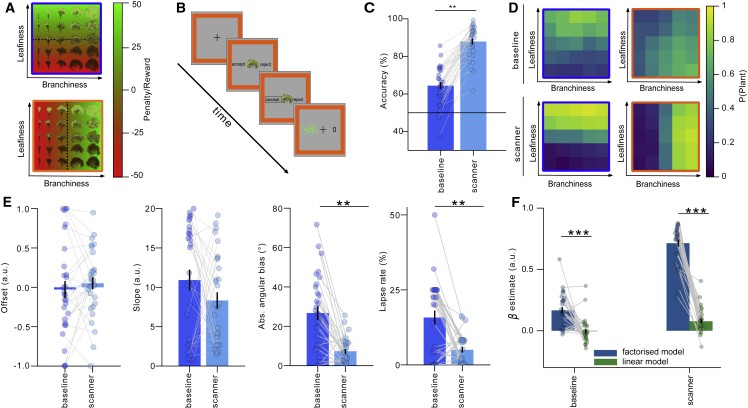
Task design and behavioral findings (A) Illustration of the 2D stimulus space. Each image shows the category boundary (dashed line) and reward or penalty (red-green color) for choosing to plant in a specific context (signaled by blue frame and orange frame). (B) Example trial sequence. Participants were asked to “accept” (plant) or reject a tree by pressing one of two buttons. Frame color signaled context. Participants received rewards and penalties for planting trees. (C) Mean accuracy improved from baseline to scan. Each dot is a participant. (D) Choice matrices show the mean probability of choosing “plant” for each tree (defined by a level of leaf and branch density) in each context for both the baseline (top) and scanner (bottom) sessions. (E) Parameters of the psychophysical model between baseline and scan: offset, slope, angular bias, and lapse rate. Each dot is a participant. ^∗∗^p < 0.01. (F) Fits of linear and factorized model at baseline and scan. Each dot is a participant. Error bars indicate SEM. See also Figure S1.

We focus on a canonical paradigm involving context-dependent classification of *D*-dimensional stimuli $x\left(i,j\right)\in R^{D}$, which vary along two underlying dimensions i and j, for which correct decisions depend on i in task A and j in task B. Healthy human participants (n = 32) categorized naturalistic (tree) stimuli, with the correct class given by “branch density” in one context and “leaf density” in the other (Figures 1A, 1B, and S1). We varied these two dimensions parametrically to generate an n-by-n grid of unique stimuli in which the density of branches and leaves were independent by design. The dimensions were *a priori* unknown to participants (Flesch et al., 2018). Accuracy increased with training, jumping from 64% ± 2% to 88% ± 2% between an initial baseline and a final test conducted in the fMRI scanner (t_29_ = 11.1, p < 0.001; Figure 1C). Using a psychophysical model to decompose errors into distinct sources, we found that this improved performance was due neither to a steepening of the psychometric curve (slope: p = 0.120), nor to a reduction in decision bias (offset: p = 0.319), although the scan session was characterized by fewer generic lapses (lapse: *Z* = −3.5, p < 0.001; Figure 1E). Instead, the fitted estimation error for the category boundary fell from 27° to 7° (angular bias: *Z* = −4.1, p < 0.001; Figure 1E). In a previous study (Flesch et al., 2018), we quantified behavioral response patterns in this trees task by fitting a model that made choices according to the two orthogonal ground truth boundaries (Flesch et al., 2018). This “factorized” model fit better than a “linear” model that learned a single boundary for both tasks, a finding we replicate here (Figure 1F; scan phase: factorized > linear t_29_ = 17.61, p < 0.0001, phase × model interaction: t_29_ = −10.84, p < 0.0001). In other words, despite having no prior knowledge of the tasks or stimulus space, participants learned over the course of training to apply the orthogonal category boundaries appropriately in each context (Figure 1D).

### The initial weight scale of a neural network controls a trade-off between learning speed and robustness

**Figure 2:**
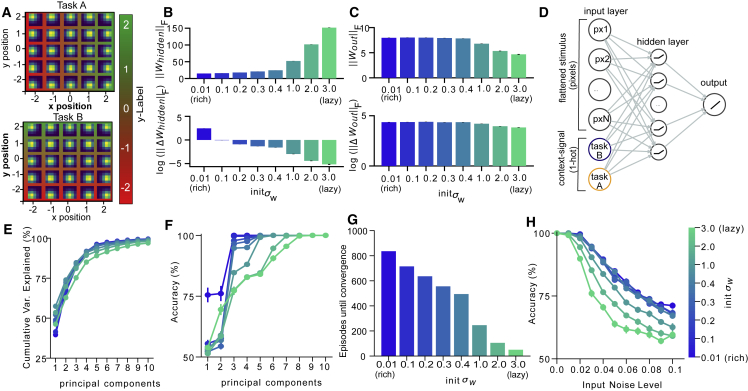
Neural network architecture and effect of weight scale on learning speed and robustness (A) In each context, the network had to predict either the x- or y-position of the mean of two-dimensional Gaussian blobs. (B) Norm of the hidden weights at convergence (upper) and overall change in weights from input to hidden layer (lower), both varied with initial weight scale (x axis and green-blue color scale). (C) Same as (B), but for hidden-to-output weights. (D) We trained a feedforward neural network with a single hidden layer of ReLU nonlinearities on the tasks. Inputs were flattened images of Gaussian blobs and a one-hot encoded context cue. (E) Variance explained after the retention of 1–10 principal components of hidden layer activity (x axis) under different initial weight scales. (F) Network accuracy as a function of retained components. Note that the rich networks (lower initial weight scale) are more robust to compression. (G) Episodes to convergence as a function of initial weight scale. Lazy networks converge faster. (H) Network performance with differing levels of input noise. Rich networks are more resilient to noise. See also Figure S2. All error bars indicate SEM.

To understand the evolution of neural codes supporting this behavior, we trained neural networks with gradient descent to perform a simplified version of the context-based categorization task. For simplicity, we replaced trees with stylized images (containing Gaussian “blobs”) that were classified according to their mean x or y coordinate in two interleaved contexts (task A and task B) and signaled to the network via unique input nodes (Figures 2A and 2D). As expected from theoretical results (Chizat et al., 2018; Woodworth et al., 2020), the norm of the weights at convergence (Figure 2B, upper) and overall change in input-to-hidden layer weights over learning (Figure 2B, lower) depended strongly on initial connection strengths (Kruskal-Wallis test on w_hidden_: H = 235.269, p < 0.0001; Kruskal-Wallis test on Dw_hidden_: H = 234.51, p < 0.0001), while the change of readout weights was substantial and differed only slightly across training regimes (Kruskal-Wallis test on w_out_: H = 199.5, p < 0.0001; Kruskal-Wallis test on Dw_out_: H = 172.077, p < 0.0001; Figure 2C). Hereafter, we refer to the extremes along the continuum of weight variances as rich (s = 0.01) and lazy (s = 3.0) regimes. Applying principal-component analysis (PCA) to the hidden layer patterns revealed that the final representations were more low-dimensional under rich learning, with just six principal components needed to explain 95% of the variance under rich learning and nine under lazy learning (Figure 2E). Critically, however, the rich regime proved more tolerant to a challenge that reduced the dimensionality of hidden unit activity: only three out of six components were needed to maintain ceiling performance, whereas eight out of nine were required under lazy learning (Figure 2F). Although learning was up to ten times faster in the lazy regime (convergence speed lazy > rich: t_29_ = 125.846, p < 0.0001; Figure 2G), the highly structured representations acquired during rich learning conferred robustness, also making performance more tolerant to the addition of Gaussian input noise (accuracy rich > lazy: t_29_ = 14.55, p < 0.0001; Figure 2H). In other words, networks initialized in the lazy regime rapidly learned to solve the task by reading out from an approximately fixed nonlinear high-dimensional random representation, whereas those initialized in the rich regime converged more slowly but exhibited strong representation learning in the input-to-hidden weights. These solutions offer complementary costs and benefits for representation learning (speed versus robustness) of task-related variables.

### Neural network simulations suggest two possible representational schemes for context-dependent decision making

**Figure 3:**
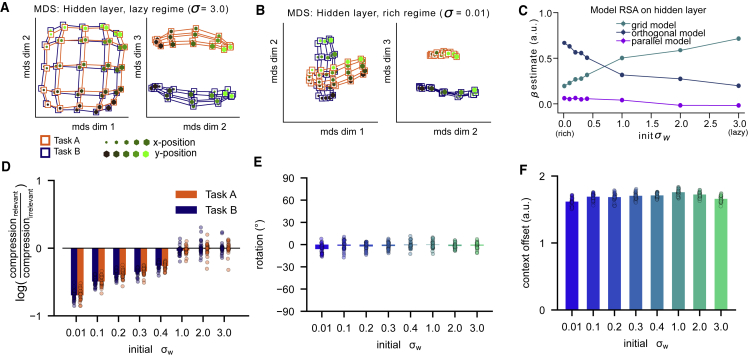
Geometry of representations in hidden layer of trained neural network (A) 3D representation of hidden layer representations for each stimulus feature (x- and y-position, dot color, and size) in each context (connecting lines, orange, and blue) after training in the lazy regime. (B) Same as (A) but for training in rich regime. Note the emergence of orthogonal manifolds that compress along the irrelevant dimension aligned with dimensions 1 and 2. (C) Fits of RDMs encoding grid, orthogonal, and parallel representational schemes to the neural network data as a function of initial weight scale. The orthogonal model (dark blue line) fits best in the rich regime, and the grid model (cyan line) fits best in the lazy regime. (D–F) Estimates for compression, rotation, and offset of the parameterized RSA model. The best-fitting RDM is characterized by parametrically varying expansion or contraction of representation on the relevant or irrelevant dimension (D), context-dependent rotation of the stimulus axes from native space into the reference frame of the response (i.e., from orthogonal to parallel model; E), and separation between contexts (F). See also Figure S6. All error bars indicate SEM.

Next, we used representational similarity analysis (RSA) and multidimensional scaling (MDS) to visualize the neural geometry of the network hidden units at convergence under either regime (Figures 3A and 3B). During lazy learning (s = 3.0) the similarity is mostly driven by the structure of the input space (including the task context) (Figure 3A); this is expected because the input weights remain close to their initial values and random high-dimensional projections approximately preserve distances between inputs (Gao et al., 2017). However, during rich learning (s = 0.01) hidden unit activity varies with context: in task A, neurons code for dimension x but not y, with the converse true for task B. In other words, task-irrelevant features were attenuated in each context, transforming the neural “grid” into two manifolds, each coding for a task-relevant dimension. Specifically, each context has a compressed and an uncompressed axis, forming a rectangle in the plane, and we hereafter call the geometry “orthogonal” when the respective compressed and uncompressed axes are perpendicular across tasks. Thus, the network learned to transform the inputs in a way that might minimize intrusions from irrelevant features in each context (Figure 3B) (Koay et al., 2019). This was confirmed by fitting model representational dissimilarity matrices (RDMs) to the hidden unit patterns at convergence (Figure S2A): a grid model that encoded the space spanned by the two feature dimensions and context fit best for lazy solutions and an orthogonal model that encoded only task-relevant dimensions along orthogonal axes fit best for rich solutions (grid model, lazy > rich: t_29_ = 29.02, p < 0.0001; orthogonal model, rich > lazy: t_29_ = 20.26, p < 0.0001; Figure 3C). Both models explained the patterns better than a parallel model that represented an encoding of the value of stimuli along two parallel planes, obtained by rotating one of the task manifolds from the orthogonal model by 90 degrees (grid model > parallel model: t_29_ = 74.69, p < 0.0001; orthogonal model > parallel model: t_29_ = 82.61, p < 0.0001, Figure 3C). We also used RSA in conjunction with a parametric model-fitting approach. Rather than fitting models encoding extremes of compression, rotation, and context separation, now we built RDMs by varying these factors continuously. Fitting this parameterized model to the neural network data confirmed that compression along irrelevant dimensions was larger under rich than lazy learning (t_29_ = 49.77, p < 0.0001; Figure 3D). The estimated rotation parameter was close to zero (Figure 3E), which suggests that information was kept in the frame of reference of the inputs, yielding orthogonal and grid-like representations in the rich and lazy regimes, respectively. In both regimes, a third dimension encoded context, indicated by a non-zero offset parameter (Figure 3F). Networks trained in the rich or lazy regime converged at different rates, suggesting that the chosen learning rate might have an impact on the representations acquired by the networks. However, repeating the simulations for a range of different learning rates revealed that while this hyperparameter choice had some impact on the weight change, the overall difference in weight changes and representations between rich and lazy learning was independent of learning rate (Figures S2B–S2D). Taken together, a simple neural network can solve the tasks either by employing high-dimensional and task-agnostic or low-dimensional and task-specific representations. The variance of weights at initialization determines how learning dynamics shape representational geometry.

### Human fMRI reveals task-specific representations consistent with those predicted by the rich training regime

**Figure 4:**
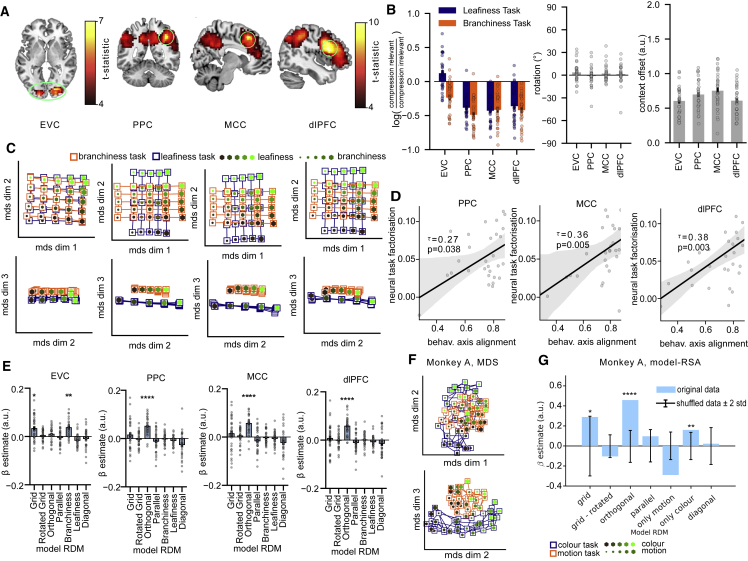
Task representations in human fMRI and macaque unit recordings (A) Results from searchlight RSA. Left: voxel regions where neural similarity patterns matched the branchiness RDM. Right: voxels where neural similarity patterns matched the orthogonal RDM. All data are corrected for multiple comparisons. (B) Data from parametric RDM fits. Error bars indicate SEM. (C) Low-dimensional projections of fMRI data from within ROIs taken from visual, parietal, and frontal regions, reconstructed from coefficients of regression model described in (B). (D) Correlation between neural task factorization (fits of orthogonal model to neural data) and behavioral axis alignment (fits of factorized model to choice matrices). Each dot is a participant. (E) Results from fitting the seven model RDMs to four independently defined ROIs, showing task-agnostic encoding in EVC and task-specific encoding in fronto-parietal areas. Error bars indicate SEM. (F) MDS projection of data from monkey A. Stimulus features are now color and motion; data from Mante et al. (2013). (G) Fits of orthogonal, grid, and control RDMs to data from monkey A. Error bars indicate ± 2 standard deviation of RSA on shuffled data (1,000 permutations). See also Figures S3 and S4. ∗p<0.05, ∗∗p<0.01, ∗∗∗p<0.001, ∗∗∗∗p<0.0001.

How, then, are task representations structured in biological brains? Our simulations furnished predictions about the neural geometry we should expect to see in BOLD data acquired during the final phase of our experiment. Univariate tests replicated standard findings, including the heightened BOLD signal in PFC on task switch relative to stay trials (Figures S3A and S3B), the correlation between BOLD signal and decision certainty in posterior parietal (Tosoni et al., 2008) and medial orbitofrontal cortex (Basten et al., 2010) (Figures S3C and S3D), and an encoding of choice value (Boorman et al., 2011) in ventromedial prefrontal cortex (vmPFC), anterior cingulate cortex (ACC), and the striatum (Figure S3E). However, to investigate neural geometry, we once again turned to a more powerful multivariate analysis of the activity patterns (RSA). We used model RDMs encoding grid, orthogonal, parallel, and various control patterns to predict brain activity using a spherical searchlight across the whole brain (Figures S2A and S4A). Crucially, we observed strong correlations with the “orthogonal” model in three major foci: the dorsolateral prefrontal cortex (DLPFC; t_30_ = 9.79, p < 0.001 corrected, peak [46 14 24]), the mid-cingulate cortex (MCC; t_30_ = 9.51, p < 0.001 corrected, peak [8 21 49]), and the posterior parietal cortex (PPC; t_30_ = 8.87, p < 0.001 corrected, peak [39 −45 45]; Figure 4A). A similar effect was observed in a left prefrontal region that the univariate analysis had revealed was sensitive to task switches, but the fit of the orthogonal model did not differ between switch and stay trials (Figure S4E). In early visual regions, neural data RDMs were best predicted by a model in which dissimilarities depended mainly on branch density (t_30_ = 6.98, p < 0.001 corrected, peak [22 −84 −3]), but no other models explained a significant amount of variance in the neural RDMs (Figure 4A). Repeating this RSA with independently defined regions of interest (ROIs) confirmed that the branchiness model fit best in early visual cortex (EVC) (Bonferroni-corrected a = 0.0071; Grid: t_30_ = 3.46, p = 0.0008; Rotated Grid: t_30_ = 0.93, p = 0.1809; Orthogonal: t_30_ = 1.76, p = 0.0442; Parallel: t_30_ = −0.75, p = 0.7692; Branchiness: t_30_ = 4.74, p < 0.0001; Leafiness: t_30_ = −3.43, p = 0.9991; Diagonal: t_30_ = −1.20, p = 0.8805; Figure 4E) whereas the orthogonal model fit best in DLPFC (Grid: t_30_ = 0.60, p = 0.2758; Rotated Grid: t_30_ = −0.19, p = 0.5746; Orthogonal: t_30_ = 8.18, p < 0.0001; Parallel: t_30_ = −1.31, p = 0.8999; Branchiness: t_30_ = 0.19, p = 0.4259; Leafiness: t_30_ = −1.59, p = 0.9388; Diagonal: t_30_ = −1.28, p = 0.8949), PPC (Grid: t_30_ = 1.31, p = 0.1007; Rotated Grid: t_30_ = −0.56, p = 0.7086; Orthogonal: t_30_ = 7.77, p < 0.0001; Parallel: t_30_ = −1.41, p = 0.9149; Branchiness: t_30_ = −0.35, p = 0.6354; Leafiness: t_30_ = −2.19, p = 0.9816; Diagonal: t_30_ = −2.73, p = 0.9947), and MCC (Grid: t_30_ = 1.08, p = 0.1448; Rotated Grid: t_30_ = 0.82, p = 0.2093; Orthogonal: t_30_ = 7.17, p < 0.0001; Parallel: t_30_ = −1.88, p = 0.9648; Branchiness: t_30_ = −0.50, p = 0.6908; Leafiness: t_30_ = −1.26, p = 0.8914; Diagonal: t_30_ = −2.18, p = 0.9815; Figure 4E). To verify that EVC encoded both dimensions irrespective of the task, whereas fronto-parietal regions employ partially compressed and orthogonal representations, we fit a support vector machine (SVM) with linear kernel and binary outputs to the relevant feature dimensions (high versus low for branchiness and leafiness) in each region and assessed the cross-validated decoding performance along the relevant and the irrelevant dimension in the same and other tasks. In all regions, decoding accuracy along the relevant dimension of the task that the decoder had been trained on was significantly above chance (Bonferroni corrected a = 0.0125; EVC: t_30_ = 8.99, p < 0.0001; DLPFC: t_30_ = 4.41, p = 0.0001; MCC: t_30_ = 5.54, p < 0.0001; PPC: t_30_ = 4.13, p = 0.0003; Figure S4C). The same dimension in the other task could be reliably decoded in EVC but not in the other regions, again suggesting that those regions attenuated irrelevant dimensions relative to the relevant ones (Bonferroni corrected a = 0.0125; EVC: t_30_ = 8.44, p < 0.0001; DLPFC: t_30_ = 2.32, p = 0.0275; MCC: t_30_ = 1.22, p = 0.232; PPC: t_30_ = 1.85, p = 0.0736; Figure S4C). To statistically compare representations in these regions, we conducted a Bayesian model comparison (RFX BMS) of linear regressions with and without the branchiness or orthogonal model RDM, fit separately to EVC, DLPFC, PPC, and MCC. Protected exceedance probabilities that quantify how likely it was that the same model explained patterns in EVC and DLPFC/PPC/MCC were extremely low (EVC and DLPFC: pep = 0.000329, EVC and MCC: pep = 0.0029, EVC and PPC: pep = 0.000191). RFX BMS within each region confirmed again that the branchiness model explained most of the patterns in EVC, while the orthogonal model yielded the best fit in DLPFC/MCC/PPC (Table S1 and Figure S4B). To summarize, in fronto-parietal areas, neural codes were largely structured as predicted by rich learning, with representations in each context projected onto orthogonal neural axes that are elongated along the relevant feature dimension and compressed along the irrelevant feature dimension. In contrast, representations in early visual areas were largely unaffected by context.

### Fronto-parietal representations in the human brain are task-specific and low-dimensional

Next, we fit the parametric RSA model to the neural data within each independently defined ROI to quantify the extent to which irrelevant information was attenuated in each context. This confirmed that in DLPFC/PPC/MCC, the neural code was compressed along irrelevant relative to relevant dimensions and remained in the naive (input) space rather than being rotated into the frame of reference of the response (“accept” versus “reject” irrespective of context) (EVC Compression Leafiness Task: z = 2.25, p = 0.0242; Compression Branchiness Task: z = 4.10, p < 0.0001; Offset: z = 4.86, p < 0.0001; Rotation: z = 1.49, p = 0.1364; DLPFC Compression Leafiness Task: z = 4.53, p < 0.0001; Compression Branchiness Task: z = 4.86, p < 0.0001; Offset: z = 4.86, p < 0.0001; Rotation: z = 1.14, p = 0.2557; MCC Compression Leafiness Task: z = 4.84, p < 0.0001; Compression Branchiness Task: z = 4.80, p < 0.0001; Offset: z = 4.86, p < 0.0001; Rotation: z = 0.27, p = 0.7838; PPC Compression Leafiness Task: z = 4.80, p < 0.0001; Compression Branchiness Task: z = 4.84, p < 0.0001; Offset: z = 4.86, p < 0.0001; Rotation: z = 0.53, p = 0.5967; Figure 4B). When we used MDS to visualize the best-fitting model RDMs for each region in three dimensions, the task-specific encoding of relevant dimensions along orthogonal manifolds in dorsal stream regions of interest can be clearly seen (Figure 4C). Finally, in neural networks rich learning is characterized by a low-dimensional neural code. Interestingly, PCA on the neural data suggested that patterns in fronto-parietal regions were higher dimensional than in EVC (number of principal components [PCs] needed to explain 95% of variance: EVC/DLPFC/PPC/MCC: 9/15/19/19; Figure S4F). However, by systematically removing components from the data using PCA on the BOLD patterns within each candidate ROI and repeating the RSA on this reduced space, we revealed that reliable correlation with the orthogonal manifolds RDM required just two components in each region of interest and that there was no measurable benefit in maintaining more than six PCs in total (Figure S4D). Similar patterns were seen for the grid model in EVC (Figure S4D). In other words, the neural representations seem to be embedded in a low-dimensional subspace focused on task-relevant stimuli, as predicted by rich learning.

### Neural task factorization predicts behavioral axis alignment

Next, we attempted to link these neural patterns to behavior. In theory, if participants had learned to filter out irrelevant dimensions, one would expect that their category judgements showed fewer signs of intrusions from those dimensions. The factorized model that was fit to human choices quantified the extent to which these were aligned with the ground truth category boundaries. This yielded an “axis alignment” score for each participant, which was correlated with the orthogonality of neural task representations across the cohort in PPC (Kendall’s tau_a_ = 0.27, p = 0.038), MCC (Kendall’s tau_a_ = 0.36, p = 0.005), and DLPFC (Kendall’s tau_a_ = 0.38, p = 0.003; Figure 4D). In other words, the category judgements of participants with more factorized neural representations respected more orthogonal category boundaries, suggesting a link between the extent to which task information is embedded in orthogonal manifolds and the ability to avoid mutual interference between tasks.

### NHP single-cell data exhibits representations as predicted by rich learning

BOLD data offers at best an indirect window on neural coding, so we additionally capitalized on a freely available dataset describing single-neuron activity in frontal eye fields (FEF) while macaques performed an equivalent context-dependent decision task on stimuli with varying color and motion coherence (Mante et al., 2013; Aoi et al., 2019). We focus on the results from monkey A because our analyses (and those reported previously) indicate that FEF neurons recorded from monkey F were only very weakly sensitive to motion even when it was decision-relevant (Aoi et al., 2019) (Figure S5D). First, we built a pseudo-population from all the recorded neurons and visualized its neural geometry in two dimensions with MDS. This revealed two orthogonal manifolds, each coding for one of the two task-relevant axes, similar to the ones observed in BOLD data and predicted by neural networks trained in the rich regime (Figure 4F). Indeed, when we fit the candidate RDMs used above to the dataset, the orthogonal RDM fit best for monkey A (grid model: p = 0.027, orthogonal model: p < 0.0001, only color model: p = 0.006; Figure 4G); an RDM coding for color alone fit best for monkey F (grid model: p = 0.004, orthogonal model: p < 0.0001, only color model: p < 0.0001; Figure S4I). Training a linear SVM on the patterns recorded in monkey A confirmed that the task-relevant, but not the task-irrelevant, dimension could be reliably decoded (same task, relevant dimension: p < 0.0001; other task, irrelevant dimension: p = 0.064; Figure S4G). We also tested dimensionality of these neural geometries using a similar PCA-based approach to the one above; the ability to decode orthogonal manifolds dropped sharply when fewer than three components were retained, suggesting that directions of highest variance were aligned with the task-relevant dimensions of context, color, and motion (Figure S4H). This analysis suggests that the orthogonal manifolds identified with the RSA lie embedded in a very low-dimensional manifold and indicates that the effect observed in human BOLD may generalize across species and recording methods.

### Task-specific representations can be achieved via non-linear gating

How does this neural coding scheme prevent interference among tasks? Early work on attention and cognitive control suggests that a gating mechanism could be employed to selectively activate units that encode information that is relevant for the task (Miller and Cohen, 2001). The classic model of cognitive control implements this gating with hard-coded biases that move activity in and out of the linear range of sigmoidal nonlinearities in the network’s hidden layer (Cohen et al., 1990). In contrast to this earlier work, our neural network is trained end-to-end on the task without enforcing the gating scheme by hand. How then do task-specific representations emerge under rich learning?

**Figure 5:**
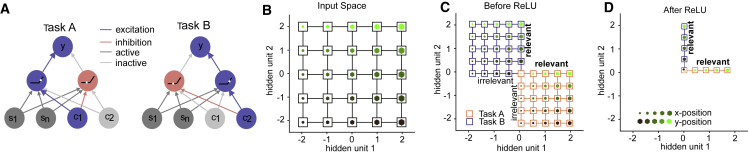
Nonlinear gating theory (A) Schematic illustration of how opposing weights from two context units leads to learning two unique subspaces. Red and blue arrows show positive and negative weights from context units, which control the sign of the net inputs in the hidden layer so that stimuli are effectively processed by different hidden units in each context. (B–D) Schematic illustration but in neural state space. (B) shows similarity structure among input stimuli with no context modulation. (C) shows the similarity structure in the hidden layer net input (before ReLU). Note the separation between contexts. (D) After the ReLU, “inhibited” (below-zero) inputs are removed, leaving two orthogonal manifolds.

We reasoned that orthogonal manifolds could emerge if the weights linking each context unit to the hidden layer were anticorrelated. Anticorrelated weights ensure that distinct subsets of hidden units are active in each context because neurons that receive negative net input in one context (and that therefore are inactive because of the rectified linear [ReLU] activation function) will receive positive net input (and be active) in the other. By wiring only the task-relevant stimulus dimension to the active population in each context, information along the irrelevant dimension is thus effectively zeroed out by the nonlinearity, creating an independent subspace for each task (Figure 5A). This would allow the network to factorize the problem, encoding the task-relevant information in a way that avoids mutual interference (Figures 5B–5D).

### Empirical evidence in neural networks and NHP recordings supports gating theory

**Figure 6:**
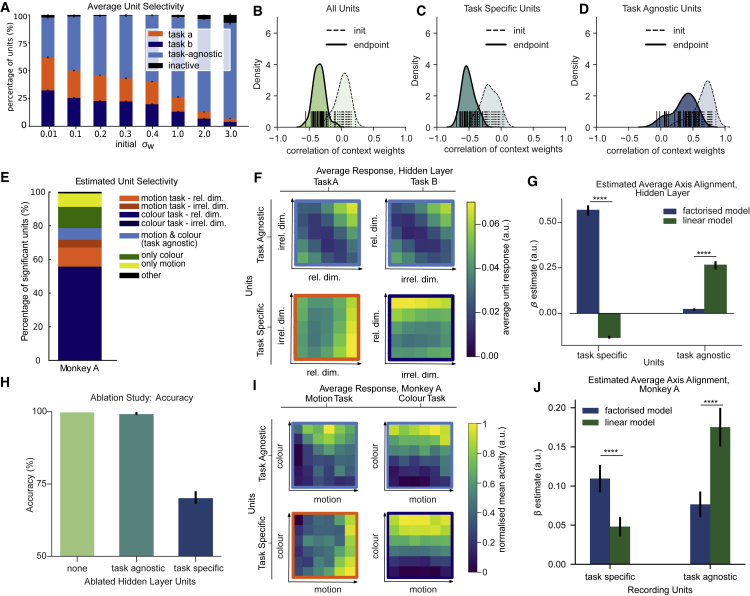
Neural network and NHP data in support of gating theory (A) Proportion of task-agnostic and task-specific units in the neural network as a function of initial weight scale. (B) Distribution of empirically observed correlation coefficients among context unit weight vectors in the neural network. (C and D) Same as (B) but separated out by “task-specific” and “task-agnostic” units as defined in (A). Note the anticorrelation in task-specific units (and overall). (E) Distribution of selectivity of single units in monkey A, using similar criteria as in (A). (F) Hidden unit selectivity for each relevant and irrelevant stimulus feature in each context. Note that task-specific units (lower panels) are mostly sensitive to relevant versus irrelevant dimension, whereas task-agnostic units code for an interaction between features. (G) Quantification of results in (F) using fits of linear versus factorized model. The factorized model fits best to task-specific units, and the linear model to task-agnostic units. (H) Results of ablation study. Ablating task-specific, but not task-agnostic, units is detrimental to performance. (I) Same as (F), but for example units from monkey A. (J) Same as (G), but for monkey A. See also Figure S5. All error bars indicate SEM. ∗p<0.05, ∗∗p<0.01, ∗∗∗p<0.001, ∗∗∗∗p<0.0001.

This theory makes several testable predictions. First, it implies that most neurons should be mixed selective, responding to combinations of stimuli and task variables. Second, however, it implies that this mixed selectivity should be structured in the rich regime, with most units in the hidden layer responding specifically to the combination of task-relevant stimulus dimension and task. Indeed, we observed that up to ∼ 60% of hidden units responded exclusively under one task or the other during rich learning (Figure 6A). Visualizing the receptive fields of the hidden layer units revealed that under rich learning, weights into task-specific units were aligned with the relevant feature dimensions, whereas under lazy learning, more heterogenous selectivity patterns were observed (Figure S5A). Investigating the magnitude of the readout weights confirmed that the network relied mostly on those task-specific units to make predictions (Figure S5B). Interestingly, when we conducted a comparable analysis for non-human primate (NHP) data, we found that the majority (∼ 65%) of significantly responsive units were also selective to either color in the color task or motion in the motion task, although there was strong bias toward the color task (Figure 6E). Third, the theory predicts that in neural networks the context weights should be anticorrelated. This is indeed the case on average in the rich regime (Figure 6B) and especially for most task-specific neurons (Figure 6C), which became anticorrelated as training progressed. In contrast, those neurons that converged to being task-agnostic were those that received strong, positively correlated input from two context units at random initialization, and this input remained positively correlated after training (Figure 6D). It thus seems likely that the initial sign of the connections from the context units to each hidden unit determines whether it is destined to be a task-agnostic or task-specific unit during training. We cannot test this in NHP data, but we can compare the response profiles of neurons defined as task-agnostic and task-specific in both model systems, revealing how their responses vary with stimulus input in either context. The theory predicts that task-specific units show a coding preference for relevant feature dimensions (with irrelevant features mapped onto units that are deactivated by the ReLU). This is exactly what is seen in both the neural network (Figures 6F–6G; factorized model > linear model: z = 4.781, p < 0.0001, d = 0.873) and the NHP data, where the responses of task-specific units are aligned to the two choice axes (Figures 6I–6J; factorized model > linear model: z = 4.643, p < 0.0001, d = 0.558). By contrast, in neural networks the remaining ∼35% of active units coded for a residual policy that collapses across both contexts (“task agnostic”), resembling the “linear” model described above (Figures 6F–6G; linear model > factorized model z = 4.781, p < 0.0001, d = 0.873). Very similar task-agnostic response patterns were observed in NHP neurons that responded significantly to stimuli but did not differentiate substantially between dimensions (Figure 6I). Just as in the neural network simulations, responses of these single units were best explained by the linear model (Figure 6J; linear model > factorized model: z = 4.033, p < 0.0001, d = 0.749). A final prediction of this theory is that, in the rich regime, performance depends critically on the task-specific neurons but not on those displaying task-agnostic selectivity. In the neural network, we thus conducted an ablation study in which the output of either the task-agnostic or task-specific neurons was set to zero at evaluation. Performance was unimpaired by the loss of task-agnostic units but dropped to ∼70% after task-specific units were removed, consistent with the use of a single linear boundary across the two contexts (Figure 6H). In contrast, under lazy learning, there was no substantial difference between ablating task-specific or task-agnostic units (Figure S5C). How did performance depend on these units? The analyses of the receptive fields suggest that under rich learning—but not under lazy learning—removing task-selective units should only impair performance on one task, not on the other task for which units were not selective. This is in fact what we observed (Figure S5C). As task-agnostic units ignored the context under rich learning, removing the task-specific units should impair performance on incongruent, but not on congruent, trials. Again, this was confirmed by our simulations (Figure S5E). We noticed a subtle congruency effect in human behavior (Figure S5F), in line with the small angular bias in their decision boundaries and the observation that irrelevant information was not fully filtered out. Together, these findings support a model of context-dependent decision-making whereby the network learns to gate information into orthogonal subsets of hidden units (of a neural network) or subspaces in PFC (of humans and NHPs) in a way that minimizes mutual interference. This scheme emerges when context input weights are anticorrelated.

## Discussion

The work described here makes three distinct contributions. The first is to formalize solutions to the learning of a canonical context-dependent classification paradigm using a feedforward connectionist (or “deep learning”) framework (Richards et al., 2019; Saxe et al., 2021). We do this by drawing upon recent work in machine learning research, which distinguishes among the learning regimes that occur when deep networks are initialized with high-variance (lazy) or low-variance (rich) weights (Rigotti et al., 2013; Fusi et al., 2016; Chizat et al., 2018; Jacot et al., 2018; Arora et al., 2019; Lee et al., 2019; Geiger et al., 2020; Woodworth et al., 2020). We derive predictions from these regimes for the context-dependent classification task, a paradigm that has been well studied before using both single-neuron electrophysiology (Mante et al., 2013; Aoi et al., 2019) and neuroimaging (Takagi et al., 2020) methods.

The second contribution is to assess these predictions using behavioral testing and functional neuroimaging in human participants and reanalysis of a dataset recorded from macaque monkeys performing an equivalent task. In humans, we find that, over the course of training, participants learned about the structure of the stimulus space and correctly inferred the orientation of the two category boundaries. After training, we observe a stylized neural geometry in the parietal and prefrontal cortices that closely matches the predictions of the “rich” regime, wherein stimuli are projected onto orthogonal subspaces on a low-dimensional manifold. A similar pattern was observed in the NHP data. Together, these data speak to a debate about whether humans and other primates learn to solve complex tasks by forming high-dimensional (and task-agnostic) or low-dimensional (and task-specific) neural codes and offer striking evidence for comparable coding principles in humans, non-human primates, and artificial neural networks.

The third contribution is an insight into the computational principles that allow the context-dependent decision task to be solved. We show that a combination of anticorrelated context inputs and ReLU (or ReLU-like) nonlinearities allows the network to effectively learn to gate task information according to context. This allows us to predict how mixed-selective neurons code for relevant and irrelevant features in both neural networks and NHPs and to anticipate the effects of silencing task-agnostic versus task-specific neurons on performance. We note that for the NHP task, where inputs arrive over time, our simple theory models the representation at late times after stimulus presentation. Adding recurrent connectivity yields a model exhibiting a “late selection” mechanism and fixed stimulus input directions across contexts, two key hallmarks identified in prior analyses (Mante et al., 2013; Aoi et al., 2019) (see Figures S5G–S5J).

There has been a recent resurgence of interest in neural networks (or “deep learning models”) as computational theories of biological brains (Richards et al., 2019; Saxe et al., 2021). A common approach is to use linear methods to examine similarities between the representations formed in biological systems (e.g., multi-neuronal or multivoxel patterns) and in the hidden units of deep networks. One corollary of our findings is that the relationship between representations formed in biological and artificial networks can critically depend on the variance of the weights at initialization. For example, when the initial weight scale is large, the similarity structure of encoded representations will closely match their input structure. This is what we saw in BOLD data from visual cortex (in our case, a more “grid-like” pattern, with higher sensitivity to variations in shape than in color). This may partly explain why previously reported improvements in model fit of trained over untrained networks tend to be relatively modest, as if the visual cortex mainly recapitulates the input data through random high-dimensional projections (Güçlü and van Gerven, 2015; Schrimpf et al., 2018).

In our data, the nature of the neural code observed in parietal and prefrontal cortex, however, was very different. Here, task-irrelevant features were compressed in each context, converting the neural “grid” into orthogonal manifolds, each coding predominantly for a task-relevant axis. This is quite striking, because conflicting reports have suggested that task-irrelevant information is retained or discarded during context-dependent decision-making (Mante et al., 2013; Takagi et al., 2020). More generally, the diverse representational structure that can emerge in the rich and lazy regimes, and its variable mapping to the brain, may shed light on why emerging representation structure can be heterogenous in trained neural networks (Mehrer et al., 2020). We note that manipulating the variance of weights at initialization should be understood as method to generate a broad range of representational geometries, rather than a model of synaptic connectivity. Crucially, instead of imposing constraints on the way in which neurons are initially connected, the same results can be achieved by adding an L2 regularizer to a network initialized in the lazy regime, which suggests that weight decay mechanisms could control whether neural populations adopt one regime or the other (Figure S4E).

Previous analyses of single-cell data from macaque PFC have emphasized that neural selectivity is mixed and representations are high dimensional, in seeming contradiction to the findings reported here (Rigotti et al., 2013; Fusi et al., 2016). One possibility is that, over prolonged training, the dimensionality of neural representations is tailored to the transfer demands of the paradigm (Musslick et al., 2017). Structured, low-dimensional representations may be favored in settings where information can be shared across tasks or stimuli, such as our trees task, where all stimuli were unique but sampled from the same underlying generative process, hence permitting generalization of latent features across tasks. By contrast, high-dimensional neural codes may emerge by preference in tasks with minimal need for generalization, such as recall and recognition of a small set of unrelated images (Barak et al., 2013; Rigotti et al., 2013). Indeed, our rich neural networks were more tolerant to degradation through compression and/or input noise than those in the lazy regime. However, the relationship between the generalization ability of the two regimes described here remains an open question. Another possibility is that training duration and/or task instructions influence representational geometry. Our participants had to infer task-relevant dimension from trial-wise feedback alone and were given extensive training prior to the scanning session. Future studies could explore whether representations consistent with lazy learning would be observed when participants were made aware of the task-relevant dimensions *a priori* and received less training.

At first glance, our findings might appear to diverge from previous analyses of the same data, in that we emphasize that irrelevant information is at least partly compressed in FEF (Mante et al., 2013; Aoi et al., 2019). However, our analysis of the NHP data focused on a relatively late epoch (300–600 ms post-stimulus). In fact, when we repeated the model-based RSA separately for early, middle, and late time windows following stimulus onset, we found that representations were more grid-like early on (encoding of both feature dimensions) but became highly task-specific in the second half of the trial (Figure S5G). Crucially, we can explain this temporal evolution of task representations with an extension of our gating theory that incorporates recurrence into the neural network model (Figure S5H). Under this account, feature-selective units keep integrating motion and color information throughout the stimulus presentation period, but the irrelevant dimension is integrated at a slower rate, giving rise to a gradual progression from grid-like to orthogonal representations. In the following delay period, the context cue continues to act as inhibitory bias on the unit encoding irrelevant features, gradually suppressing its activity just enough so that by the time of a response, only task-relevant information is preserved, leaving a fully orthogonal and task-specific representation (Figure S5I). When we visualized the geometries separately for early, middle, and late windows within the stimulus interval, we observed a similar temporal evolution from grid-like to more orthogonal representations in both the recurrent neural network and monkey recordings (Figure S5J). Notably, similar evolutions of representational geometries over the time course of a trial have recently been reported in the context of working memory tasks (Panichello and Buschman, 2021).

Another recent paper has emphasized that the neural geometry for distinct tasks in macaque PFC can become aligned along parallel manifolds, with representations for common action or outcome associations aligned in neural space (Bernardi et al., 2020). An equivalent effect in our paradigm would be that tree representations are rotated into a frame of reference of “plantworthiness”—whether the tree should be accepted for planting or not—which we tested with a “parallel model” RDM but failed to find evidence for in either neural data or the network hidden units. One important difference in our work is that, in order to separate decision and motor activity, in the fMRI study we varied the motor contingencies from trial to trial, meaning that there is no real benefit to representing the decision directly in the response frame in our task. In fact, further neural network simulations revealed that, in a two-layer neural network, orthogonal representations dominated in the first hidden layer, but more parallel representations could be enforced in the subsequent layer, more consistent with the findings of Bernardi et al. (2020) (Figures S2F–S2I). We take this to imply that in a task where response contingencies were not randomized from trial to trial, we might see parallel representations emerge in a putative downstream stage—for example, premotor cortex—but this contention remains to be tested.

Our simple neural network model and Gaussian “blob” tasks introduced a considerable gap in model and data complexity between our simulations and the fMRI experiment with human participants. One concern that remains is whether our findings generalize to more complex model architectures and datasets. To address this, we trained deep convolutional neural networks (CNN) directly on the trees task and investigated the representations formed in the hidden layers under rich and lazy learning (Figure S6A). This confirmed that rich learning induces highly structured representations that progressively transform the inputs from grid-like representations in the early layer to orthogonal representations in the intermediate layer to parallel representations in the deep layers (Figure S6B), whereas under lazy learning, all hidden layers exhibited task-agnostic representations (Figure S6C). Future work could investigate how the context signal is extracted from visual information alone and used to guide feature selection in these more complex architectures.

On a related note, it remains unclear how the context signal could be implemented in the biological brain. The CNN simulations suggest that a feedforward architecture can extract context cues even if they are just presented as visual cues (colored rectangles), in contrast to individual one-hot units. In reality, however, the notion of “context” is much richer, as it can be provided by either visual or auditory cues and either explicitly via instruction or implicitly via temporal statistics of the training curriculum. While our work suggests how context signals could be used to form task-specific representations, the mechanisms that extract these signals from percepts and utilize them to sculpt representational geometry are avenues for future work.

This concern speaks to a broader question of how the human brain uses context to partition experiences and how the specific training regime we subjected our participants to shapes neural representations. In previous work, we demonstrated that humans learn better under blocked curricula, where one task is learned at a time, compared to interleaved curricula where both tasks are randomly interspersed (Flesch et al., 2018). For the fMRI study, we subjected our participants to a blocked curriculum. Future work would be required to investigate how the temporal statistics of training samples shape the geometries of task representations.

Taken together, our findings suggest striking similarities between representations of task rules in biological and artificial neural networks. It indicates that for context-dependent decision tasks learned sequentially via trial and error, the human brain appears to utilize a coding scheme that minimizes representational overlap between these tasks, like the one adopted by a neural network trained in the rich regime on interleaved data.

## STAR★Methods

### Key resources table

**Table:** 

REAGENT or RESOURCE	SOURCE	IDENTIFIER
**Deposited data**		

Human Behavioral Data	This paper	https://www.doi.org/10.17605/OSF.IO/7VCGH
Human fMRI data (preprocessed)	This paper	https://www.doi.org/10.17605/OSF.IO/7VCGH
Neural Network data	This paper	https://www.doi.org/10.17605/OSF.IO/7VCGH

**Software and algorithms**		

Custom code for Simulations, Experiments and Data Analysis	This paper	https://doi.org/10.5281/zenodo.5807610
Python 3.8.5	Python	https://www.python.org/; RRID: SCR_008394
MATLAB R2019b	Mathworks	https://uk.mathworks.com/products/matlab.html; RRID: SCR_001622
Psychtoolbox 3	Mario Kleiner, David Brainard, Denis Pelli, Chris Broussard, Tobias Wolf, Diederick Niehorster	http://psychtoolbox.org/; RRID: SCR_002881
VBA Toolbox	Jean Daunizeau, Lionel Rigoux	https://mbb-team.github.io/VBA-toolbox/
SPM12	Wellcome Trust, London	https://www.fil.ion.ucl.ac.uk/spm/software/spm12/; RRID: SCR_007037
SNPM 13	Tom Nichols	http://www.nisox.org/Software/SnPM13/; RRID: SCR_002092
Numpy 1.19.2	Community Project	https://numpy.org/; RRID: SCR_008633
Scipy 1.6.1	Community Project	https://scipy.org/; RRID: SCR_008058
Scikit-Learn 0.24.2	Community Project	https://scikit-learn.org/stable/; RRID: SCR_002577
Pytorch 1.9	Facebook	https://pytorch.org/; RRID: SCR_018536
Tensorflow 1.2	Google	https://www.tensorflow.org/; RRID: SCR_016345
Nibabel 3.2.1	NiPy	https://nipy.org/packages/nibabel/index.html; RRID: SCR_002498

### Resource availability

#### Lead contact

Further information and requests should be directed to and will be fulfilled by the Lead Contact, Timo Flesch (timo.flesch@psy.ox.ac.uk)

#### Materials availability

The study did not generate any new materials.

### Experimental model and subject details

#### Human participants

A total of 32 participants (mean age 24.44y, 31 right-handed, 21 female) with no history of neurological or psychiatric disorders were recruited from a participant pool at the University of Granada. One participant was excluded from the analysis due to equipment failure during the scanning session, leaving 31 participants for the fMRI analysis. For another participant training data was not recorded due to disruption of their internet connection, leaving 30 participants for all behavioral analyses. All participants gave written informed consent prior to taking part in the study. The experiment received approval from the ethics board of the University of Granada. Participants were compensated for their time with 38€. The experiment consisted of several sessions completed on three successive days (Figure S1A). All participants completed a pre-screening study on day 1 that assessed their eligibility for the main experiment. The main experiment consisted of a browser-based training session on day 2, and a refresher and scanning session on day 3, which took place at the fMRI institute of the University of Granada.

#### Nonhuman primate data

NHP results were based on a reanalysis of data recorded from monkey frontal eye fields (FEF) during performance of comparable context-based decision-making tasks. These data have already been intensively scrutinised in past work (Mante et al., 2013; Aoi et al., 2019). In the experiment, two monkeys were asked to discriminate between distinct levels of motion direction and color of random dot stimuli, with only one dimension being relevant in each context, just as in our experiments. Stimuli spanned a similar 2D grid (motion directions varying from left to right, color gradient from green to red) as our trees and Gaussian blobs. Further details are available in ref (Mante et al., 2013).

### Method details

#### Human behavioral / fMRI experiment

##### Stimuli

Participants performed a virtual gardening task for which they had to discover rules that determined growth success of tree stimuli in two different gardens. Trees were generated by in house-code and were built to vary parametrically in five discrete steps along two different dimensions, the density of leaves (“leafiness”) and the density of branches (“branchiness”), yielding 25 unique class. We generated multiple stimuli per level of leafiness and branchiness and sampled these exemplars randomly without replacement for training and test sessions at the level of individual participants so that no physical stimulus was presented twice during the experiment.

##### Pre-screening session (Day1)

We previously showed that learning is mediated by an *a priori* tendency to factorise tree space into dimensions of leafiness and branchiness (Flesch et al., 2018). To measure this prior in our participants we first used an online task in which participants moved tree exemplars within a circular open arena via drag and drop on the screen, attempting to arrange them so that distance between trees was proportional to their perceived dissimilarity (Figure S1B). Participants completed six arrangement trials of 25 trees, with trees sampled from the whole 5x5 grid of branchiness and leafiness on each trial. At the beginning of each trial, the trees were randomly arranged in an attempt to minimize other sources of bias. The allocation of exemplars to trials was randomized across subjects. We correlated the dissimilarity matrices derived from the arrangements with a model matrix that represented a perfect grid-like arrangement to compute a “grid score” for each participant. We planned to exclude participants who failed to reach the median grid score reported in the previous study where participants were recruited online (Flesch et al., 2018), but no participants met this criterion (Figure S1C).

##### Training session (Day2)

On day 2, participants took part in an online training session in which they learned to perform the task. On each trial participants first viewed a cue indicating the context (or “garden”), which was a blue or orange rectangular frame presented for 1000ms. Next, a tree was displayed for 1500ms within the frame, together with the response contingencies (“plant” or “don’t plant”) which were indicated by left and right arrow buttons on either side of the tree stimulus. These contingencies (i.e., whether “plant” was mapped onto the left or right button) were varied randomly from trial to trial. The stimulus and response interval were always set to 1500ms. A response provided within this interval was highlighted by a rectangle drawn around the chosen option (“plant” versus “don’t plant”). Participants were asked to learn to plant trees that grew successfully. Tree growth success depended on leafiness in one context and branchiness in another and was signaled by a numerical reward, ranging in five steps from −50 to +50. For example, for a given participant, trees occurring within the orange frame might grow successfully if they had fewer leaves, whereas trees occurring within the blue frame might grow successfully if they had more branches. Feedback, where available (see below) was presented for a period of 500ms (800ms for missed trials) and consisted of a numerical reward (if the tree grew successfully) or penalty (if it did not) for planting a tree, and always a reward of zero for not planting a tree. At the beginning of the feedback period, the tree stimulus was replaced by a fixation cross and the response contingencies were replaced by numeral rewards. These rewards/penalties were mapped onto the relevant dimension (branchiness/leafiness) and hence varied in five discrete steps from −50 to +50. Rewards (values above 0) were displayed in green, whereas penalties (rewards below zero) were displayed in red. Rewards of zero were displayed in black. Again, the chosen option was highlighted by a rectangle, with its color matching the color of the reward value (red/green/black). For training sessions, the intertrial interval (ITI) had a duration of 1000ms. The directionality of the rewards (more versus less leafy/branchy trees grow better) and the task order during the main training phase were fully counterbalanced across participants.

The training session consisted of three different blocks in which contexts could be either *blocked* or *interleaved*. *Blocked* means that all trials of one context were presented first, followed by all trials in another context, with the order counterbalanced over participants. *Interleaved* means that trials were shuffled so that they occurred in random order, but with exactly the same number in each context. Participants underwent a brief interleaved familiarisation phase with feedback (50 trials), followed by an interleaved baseline test (200 trials, no feedback). There was then a long main training session which was blocked (900 trials) (Figure S1A). The purpose of the baseline training and test was to familiarise the subjects with the task and to assess the effectiveness of the main training.

##### Scanning session (Day3)

The test session consisted of a brief refresher phase (interleaved, 50 trials, feedback) and the main test phase (interleaved, 600 trials, no feedback). The refresher was completed on the experimenter’s laptop and was identical to the baseline training on day 2. For the test phase inside the scanner, we used a jittered ITI of 2000-6000ms (uniform) during which only the gray background was displayed. The total length of all ITIs was restricted such that all runs had equal length.

##### fMRI acquisition

Magnetic resonance images were recorded with a 3T Siemens scanner with a 32-channel head coil. A high-resolution T1-weighted structural image (voxel size = 1x1x1 mm, 176x256x256 grid, TR = 1900ms, TE = 2.52ms, TI = 900ms) was acquired for each participant prior to the task. Each fMRI image contained 32 axial echo-planar images (EPI) in descending sequence (3.5x3.5x3.5mm isotropic, slice spacing 4.2mm, TR = 2000ms, flip angle = 80, TE = 30ms). We collected fMRI data in six independent runs of 345volumes each.

##### fMRI pre-processing

Pre-processing was conducted in MATLAB with SPM12 and custom scripts. For each participant, functional scans were first realigned to the first scan. As EPIs were acquired in descending sequence, we applied a slice time acquisition correction with the middle slice (TR/2 = 1 s) as reference. Next, the structural scan was co-registered to the mean EPI. Anatomical scans were normalized to standard Montreal Neurological Institute (MNI) 152 template. EPIs were normalized to the template using tissue probability maps for gray matter, white matter, and cerebrospinal fluid. The EPIs were resliced to 3x3x3mm resolution. For univariate analyses, we applied smoothing with a full width half maximum (FWHM) Gaussian kernel of 8mm.

#### Neural network simulations

The simulations were implemented and results analyzed in Python using the NumPy, SciPy, Pytorch and Scikit-Learn packages. Due to the simplicity of the architecture, gradients and optimization procedures for the simple feedforward MLPs were derived by hand and implemented in raw NumPy. The more complex simulations with auxiliary RDM loss were implemented in Tensorflow.

##### Task design

We replaced the fractal tree images with two-dimensional isotropic Gaussian “blobs.” The stimulus space was spanned by parametric modulation of the x and y coordinates of these blobs in five discrete steps. Inside this 5x5 grid, neighboring blobs were partially overlapping, allowing the network to infer similarity structure based on co-activation of input units. We used a similar context-dependent decision-making task as for our human participants. There were two contexts, in each of which only one feature dimension (either the x- or y-location) was diagnostic of the correct output (the other being an irrelevant dimension) and mapped onto a numerical reward ranging from −2 to 2. The network was trained to predict the reward received in each situation. To assess performance and representational geometries, we fed trials covering all combinations of the two feature dimensions (x/y location) and context into the network and recorded hidden layer activity patterns as well as network outputs for each stimulus.

##### Neural network architecture

Our model was a feed-forward network architecture with a single hidden layer. Input units encoded pixel intensities of vectorised and normalized images of Gaussian blobs. Each image had a down-sampled resolution of 5x5 pixels, hence resulting in 25 stimulus input units. Two additional one-hot encoded inputs (1 or 0) signaled the context to the network. All 27 inputs were projected into a hidden layer with 100 units, which were in turn passed through Rectified Linear Unit (ReLU) nonlinearities. The hidden units projected onto a single linear output unit.

##### Weight initialisation

All network parameters were initialised with random draws from Gaussian distributions with a mean of zero. To control whether the network operated in the rich or lazy regime, we modified the variance of these distributions systematically, ranging from 0.01 (rich regime) to 3 (lazy regime). We call this “initial weight scale” in the main text. These values were derived empirically by observing their impact on the relative change of the weight norm and shape of the loss trajectories during training. Weights to the output unit were instead initialised with a variance scale of 1/ $\sqrt{n_{h}}$ where $n_{h}$ is the number of hidden units. All biases were initialised to zero.

##### Training

We collected 30 independent runs (unique random initialisations) per initial weight scale condition. On each run, the network was trained with minibatch gradient descent (batch size 50, interleaved data, learning rate 0.001, SGD optimizer) on 10000 iterations. The model was trained on the Mean-Squared-Error (MSE-Loss) between the true and predicted reward associated with each stimulus:

$$J\left(W\right)=\frac{1}{n}\sum_{i=1}^{n}{\left(y_{i}-f\left(x_{i},W\right)\right)}^{2}$$

##### Addition of Gaussian input noise

We investigated the robustness of different training regimes to additive Gaussian noise in the inputs. The model architecture and training procedures were identical to the ones described above. Again, we collected 30 independent runs per weight scale, ranging from 0.01 to 3 in eight steps. However, this time, we added Gaussian noise drawn from a standard normal distribution to the input units at test. The strength of this noise was varied parametrically in 10 steps from 0 to 0.1, allowing us to investigate the impact of different noise levels on performance.

##### Impact of learning rate

We tested whether the learning rate push the network into either the rich or lazy regime. Starting from a lazy (weight scale 3) or rich (weight scale 0.01) initialisation, we trained the network with 20 independent runs per learning rate, which ranged from 0.001 to 0.01 in 10 steps. The values were chosen to ensure that learning dynamics remained stable.

##### Controlling the learning regime via L2-regularization

We investigated whether a network initialised in the lazy regime could be pushed into the rich regime by adding a regularisation term that favored small weights. For this, we added an L2 regulariser to the loss function:

$$J\left(W\right)=\frac{1}{n}\sum_{i=1}^{n}{\left(y_{i}-f\left(x_{i},W\right)\right)}^{2}+\lambda \;\|W{\|}_{2}^{2}$$

The hyperparameter λ controlled the regularisation strength. We initialised the network in the lazy regime (weight scale 3) and collected 30 independent runs per regularisation strength, which ranged from 0 to 0.1.

#### Controlling the hidden layer representations via auxiliary loss function

To test whether a network with a single hidden layer could in principle learn a parallel representation, we carried out a new set of simulations in which we introduced an auxiliary loss function that controlled hidden layer representations. On each training step, a minibatch of all 50 stimulus types was passed into the network. In the hidden layer, this yielded a 100x50 activity matrix $Y_{hidden}$. We computed a 50x50 RDM from these activity patterns (Euclidean distance) as follows:

$$G=Y_{hidden}^{T}Y_{hidden}$$

$$RDM_{hidden}=diag\left(G\right)+diag{\left(G\right)}^{T}-2G$$

We then calculated the mean squared error between this RDM and a target RDM, which was chosen to be either the grid, orthogonal or parallel model RDM described above. The total loss of the network was a weighted sum of the standard supervised objective (the MSE between the network’s output and the target label) and this RDM loss:

$$J\left(W\right)=\frac{1}{n}\sum_{i=1}^{n}{\left(y_{i}-f\left(x_{i},W\right)\right)}^{2}+\frac{\beta }{2}{\left(vec\left(RDM_{target}\right)-vec\left(RDM_{hidden}\right)\right)}^{2}$$

This encouraged the network to learn the task while being pressured to acquire the representation imposed through the RDM loss. We performed a random search to find hyperparameters that allowed the network to learn the task with a parallel representation in the hidden layer. As this was unsuccessful, we then introduced a second hidden layer (100 ReLUs) to the network to increase its capacity and repeated the procedure with the RDM loss applied to the second layer.

#### Convolutional neural network simulations

To test whether the findings reported with a small MLP generalize to more realistic settings, we trained a deep convolutional neural network (a variant of AlexNet) on the trees task. The network received the same tree images (with orange/blue frame to signal the context) as inputs as the human participants.

##### Dataset and task design

The dataset consisted of 20000 training and 10000 test RGB images (96x96x3 pixels) of fractal trees, pasted on a gray background and surrounded by an orange rectangle in context A, or blue rectangle in context B. Just like in the original trees task, the appearance of the trees was varied parametrically, spanning a 5x5 grid of different levels of branchiness and leafiness. In each context, the network was tasked to predict the level of the relevant feature dimension, just like in the MLP simulations explained above.

##### Architecture

The CNN is a variant of AlexNet and consists of a series of five convolutional layers with/without max-pooling (1^st^ Conv2d: 64 filters, size 11, stride 4, padding 2, ReLU, max-pooling size 3, stride 2; 2^nd^ Conv2d: 192 filters, size 5, padding 2, ReLU, max-pooling size 3, stride 2; 3^rd^ Conv2d: 384 filters, size 3, padding 1, ReLU; 4^th^ Conv2d: 256 filters, size 3, padding 1, ReLU, 5^th^ Conv2d: 256 filters, size 3, padding 1, ReLU) followed by a fully-connected layer (512 units, ReLU) and a single linear readout unit.

##### Training procedure

The CNN was trained with minibatch gradient descent (batch size 128) for 200 epochs. We used an Adam Optimizer (learning rate 1e-4). We trained the network in the lazy and rich regime by changing the variance of weights and hence their overall norm at initialisation. For each regime, we collected 30 independent training runs.

#### Recurrent neural network extension

Let $x_{1}\left(t\right)\in \left[-1,1\right]$ be the signed motion coherence over time in a trial, and $x_{2}\left(t\right)\in \left[-1,1\right]$ be the signed color level over time, which can be stacked into the column vector input $x\left(t\right)={\left[x_{1}\left(t\right)\;x_{2}\left(t\right)\right]}^{T}$. Let $u\left(t\right)\in R^{2}$ be the task context input encoded as a one hot vector (+1 in the first element for context A, +1 in the second element for context B).

The network contains four neuron classes, and the overall architecture is depicted in Figure S7B. In particular, these comprise a pair selective for positive/negative motion and task, and an pair selective for positive/negative color and task. Each neuron receives stimulus input through the input-to-hidden weights:

$$W_{x}=\left[\begin{array}{ll} 1 & 0\\ -1 & 0\\ 0 & 1\\ 0 & -1 \end{array}\right]$$

Each neuron class also receives task input, with the motion neurons receiving inhibitory input in the color task and the color neurons receiving inhibitory input in the motion task. The task-to-hidden weights are

$$W_{u}=\left[\begin{array}{ll} 0 & -w\\ 0 & -w\\ -w & 0\\ -w & 0 \end{array}\right]$$

where w is a parameter controlling the strength of context-driven inhibition.

The network has recurrence, which we assume has an autapse structure such that each neuron has self recurrence with weight one to enable persistent activity.

We emphasize that all four neuron classes are mixed selective, in the sense that their response depends on a combination of stimulus and task. However, this mixed selectivity is not random, rather it is highly structured.

The neural activity dynamics are given by the standard firing rate equations

$$\frac{d}{dt}h\left(t\right)=-h\left(t\right)+f\left(h\left(t\right)+W_{x}x\left(t\right)+W_{u}u\left(t\right)\right)$$

where f(⋅) is the firing rate nonlinearity, which here we take to be the ReLU function (f(v)=max{v,0}).

Finally the output of the network r is computed through readout weights $W_{o}=\left[1\;-1\;1\;-1\right]$, i.e., by summing or subtracting the relevant hidden unit activity,

$$r\left(t\right)=W_{o}h\left(t\right)$$

##### Input dynamics

We now describe the temporal structure of a trial. We assume that between trials, neural activity resets such that we have the initial condition h(0)=0. We assume that input stimuli arrive with a temporal profile $p_{x}\left(t\right)$ that is rescaled by the motion coherence m and color coherence c, such that the input is

$$x\left(t\right)=\left[\begin{array}{l} mp_{x}\left(t\right)\\ cp_{x}\left(t\right) \end{array}\right]$$

For simplicity we take $p_{x}\left(t\right)=ae^{-t/ô}+b$ for $0\;<\;t\;<\;t_{x}$, and $p_{x}\left(t\right)=0$ otherwise, to reflect a sharp onset transient followed by decay to a steady state.

The context signal arrives with a temporal profile $p_{u}\left(t\right)$, turning on with the stimulus and remaining on during the delay period until some time $t_{u}>t_{x}$. For simplicity we take $p_{u}\left(t\right)$ to be a pulse (one for times between 0 and $t_{u}$, zero otherwise). Let z be 1 in the motion context and 0 in the color context. Then we have

$$u\left(t\right)=\left[\begin{array}{l} zp_{u}\left(t\right)\\ \left(1-z\right)p_{u}\left(t\right) \end{array}\right]$$

### Quantification and statistical analysis

#### Human behavioral/fMRI data

##### Psychophysical model of human choices

To quantify sources of error in the choice patterns, we fit a psychophysical model to the choices of each participant. The model assumed that each tree was categorised with respect to a linear category boundary in tree space, via a logistic choice function. The model comprised four free parameters: (1) angle of the decision boundary in tree space (the boundary was assumed to always pass through the center of the 2D space), (2) a decision bias or offset to the inflection point of the logistic function; (3) the slope of the logistic function (iv) a proportion of random lapses. The model is identical to that in ref (Flesch et al., 2018) where it is described in more detail. From the estimated category boundary, we calculated an angular bias, quantifying the absolute disparity between the estimated and ground-truth task-specific category boundaries. The model was fitted to human choice by minimizing the difference between empirical and predicted choice patterns.

##### Group level inference

Inference was performed via paired t tests or signed-rank tests when valuations of the assumptions of t tests were observed.

##### fMRI data analysis: GLMs

Data were analyzed using SPM12, the RSA toolbox (Nili et al., 2014) and custom scripts written in MATLAB. We used a general linear model (GLM) approach for all univariate analyses. A 128 s temporal high-pass filter was applied to remove low-frequency scanner artifacts. Temporal autocorrelation was estimated with a first-order autoregressive model (AR-1). All GLMs contained regressors coding for onset and duration (boxcar until participant response) of events, which were convolved with the canonical haemodynamic response function (HRF). Six motion parameter estimates from the pre-processing stage were included as nuisance regressors in all GLMs. Each run was represented by a separate set of regressors in the GLM, and run number was encoded by a dummy variable. Observed fMRI data at single subject level was regressed against this design matrix. Our analyses are based on four different GLMs. The first GLM (GLM1) had two predictors of interest (task switch trials and task stay trials), locked to cue onset. GLM2 included two parametric regressors of absolute distance of stimuli to the category boundary, for the relevant and irrelevant dimension, respectively. GLM3 included parametric regressors of the stimulus value and their interaction with choice. GLM4 was constructed for representational similarity analysis (RSA) and fitted to unsmoothed EPIs. It had 50 regressors per run, one for each combination of context (“north garden”/blue rectangle versus “south garden”/orange rectangle), branchiness (1 to 5) and leafiness (1 to 5).

##### Representational similarity analysis of human fMRI

GLM4 (described above) was fit to neural data at single-voxel level. We then constructed neural Representational Dissimilarity Matrices (RDMs) using a spherical searchlight (radius 12mm). For each searchlight sphere, we computed cross-validated neural RDMs from the condition-by-voxel matrix of estimated neural responses using Pearson correlation distance between pairs of conditions from distinct runs:

$$d\left(x_{i},x_{j}\right)=1-\frac{\left(x_{i}-{\bar{x}}_{i}\right){\left(x_{j}-{\bar{x}}_{j}\right)}^{T}}{\sqrt{\left(x_{i}-{\bar{x}}_{i}\right){\left(x_{i}-{\bar{x}}_{i}\right)}^{T}\left(x_{j}-{\bar{x}}_{j}\right){\left(x_{j}-{\bar{x}}_{j}\right)}^{T}}}$$

This yielded a 300x300 RDM (50 conditions per run, six runs). All analyses excluded within-run similarity data (e.g., blocks of 50 conditions on the major diagonal). We constructed seven model RDMs to probe for the existence of task-related representational geometries in the fMRI activity patterns: the (1) grid model, (2) orthogonal manifold model, (3) parallel manifold model and (4) rotated grid model, (5) only branchiness model, (6) only leafiness model and (7) diagonal model. Let the vectors of branchiness and leafiness be $b={\left[-2,-1,0,1,2\right]}^{T}$ and $l={\left[-2,-1,0,1,2\right]}^{T}$. Let the task vector be defined as $t={\left[0,1\right]}^{T}$. Let the matrix of all possible ordered tuples of context, branchiness and leafiness be:

$$X^{50x3}=\left\{\left(x,y,z\right):\;x\in t,y\in b\;\mathrm{and}\;z\in l\right\}$$

The first model encoded two parallel, evenly spaced grids (unit distance), representing each combination of context, branchiness and leafiness. This RDM was constructed by computing all pairwise Euclidean distances between the rows in X. The second model was obtained by taking the grid model and projecting stimuli onto the task-relevant axes for each context.

Let $X_{A}$ be the submatrix for the first task, i.e., where ti = 0 and $X_{B}$ the submatrix for the second task, i.e., where ti = 1:

$$X_{\mathrm{grid}}=\left[\begin{array}{l} X_{A}\\ X_{B} \end{array}\right]$$

Let $Y_{A}$ be the projection matrix for the first task and $Y_{B}$ the projection matrix for the second task:

$$Y_{A}=\left[\begin{array}{lll} 1 & 0 & 0\\ 0 & 0 & 0\\ 0 & 0 & 1 \end{array}\right]$$

$$Y_{B}=\left[\begin{array}{lll} 1 & 0 & 0\\ 0 & 1 & 0\\ 0 & 0 & 0 \end{array}\right]$$

Then, the orthogonal model would be obtained by stacking $X_{A}Y_{A}$ and $X_{B}Y_{B}$:

$$X_{orth}=\left[\begin{array}{l} X_{A}Y_{A}\\ X_{B}Y_{B} \end{array}\right]$$

The RDM was obtained from this coordinate matrix by computing the pairwise Euclidean distances between its rows. Thus, for each context, stimuli differed along the task-relevant dimension (unit distance), and representations of different tasks were orthogonal to each other. The third model was obtained by rotating one of the task vectors from the second model by 90 degrees, considering the reward assignment the participant had been trained on (hence discriminating “plantiness” of trees, i.e., the extent to which “plant” was the correct answer). For example, if higher feature values led to larger rewards in both contexts, the rotation matrix would be defined as:

$$R_{A}\left(90\right)=\left[\begin{array}{lll} 1 & 0 & 0\\ 0 & cos\left(90\right) & sin\left(90\right)\\ 0 & -sin\left(90\right) & cos\left(90\right) \end{array}\right]$$

And the model would be obtained by stacking $X_{A}Y_{A}R_{A}$ and $X_{B}Y_{B}$ and computing pairwise Euclidean distances between its rows:

$$X_{par}=\left[\begin{array}{l} X_{A}Y_{A}R_{A}\\ X_{B}Y_{B} \end{array}\right]$$

For the fourth model, we performed the same rotation on the grid model, by stacking $X_{A}R_{A}$ and $X_{B}$:

$$X_{rotgrid}=\left[\begin{array}{l} X_{A}R_{A}\\ X_{B} \end{array}\right]$$

The fifth and sixth models served as controls, based on the assumption that early visual areas might exhibit task-agnostic shape (branchiness) or color (leafiness) sensitivity. These models were obtained by projecting both task-specific submatrices either onto the branchiness $\left(X_{A}Y_{A}\;and\;X_{B}Y_{A}\right)$ or onto the leafiness dimension $\left(X_{A}Y_{B}\;and\;X_{B}Y_{B}\right)$.

$$X_{branch}=\left[\begin{array}{l} X_{A}Y_{A}\\ X_{B}Y_{A} \end{array}\right]$$

$$X_{leaf}=\left[\begin{array}{l} X_{A}Y_{B}\\ X_{B}Y_{B} \end{array}\right]$$

The last model was obtained by taking the grid of all combinations of branchiness and leafiness and projecting trees onto the main diagonal, ranging from low leafiness and low branchiness to high leafiness/branchiness, with the projection $XP^{T}\;where:$

$$P=\left[\begin{array}{ll} 1 & 0\\ 0 & cos\left(45\right)\\ 0 & sin\left(45\right) \end{array}\right]$$

so that:

$$X_{diag}=X_{grid}P^{T}$$

This last model was based on the competing hypothesis that humans may have ignored context and optimized for a strategy that yielded 70% correct on both tasks (Flesch et al., 2018). Within a given structural ROI or searchlight sphere, we repeated the 50x50 model RDMs over runs to match the 300x300 neural RDMs, setting the within-run-dissimilarities to NaN. We then regressed z-scored vectorised neural RDMs against z-scored sets of vectorised model RDMs using a multiple linear regression at single subject level:

$$RDM_{brain}=\beta_{0}+\beta_{1}\;RDM_{grid}+\beta_{2}\;RDM_{rotatedgrid}+\beta_{3}\;RDM_{orth}+\beta_{4}\;RDM_{parallel}+\beta_{5}\;RDM_{branch}+\beta_{6}\;RDM_{leaf}+\beta_{7}\;RDM_{diag}$$

Statistical inference was performed with a group-level t test of the regression weights against zero. Correction for multiple comparisons was conducted via non-parametric cluster correction as implemented in the SNPM toolbox (FDR threshold < 0.05). To avoid circular inference, all post hoc visualizations and analyses within ROIs were performed in leave-one-subject-out cross-validated ROIs derived from the activity peaks identified with the searchlight approach (12 mm radius).

##### fMRI RSA: parameterized model

In order to obtain more fine-grained estimates of the neural geometry, we also fit a parameterised model to the cross-validated ROIs identified with the searchlight approach. We constructed a space of model RDMs by varying six parameters, one controlling the angle between the task-specific grids (which rotated one task manifold by up to 90 degrees in either direction, so that representations could be orthogonal, parallel, antiparallel or anything in between), four controlling for the compression of relevant and irrelevant dimensions within each context, and one controlling for the separation of contexts. Let the vectors of branchiness and leafiness be $b={\left[-2,-1,0,1,2\right]}^{T}$ and $l={\left[-2,-1,0,1,2\right]}^{T}$. Let the task vector be defined as $t={\left[0,1\right]}^{T}$. Let the matrix of all possible ordered tuples of context, branchiness and leafiness be:

$$X^{50x3}=\left\{\left(x,y,z\right):x\in t,y\in b\;and\;z\in l\right\}$$

This matrix consists of two 25x3 blocks, one for each task, each of which encodes the levels of the context signal, branchiness and leafiness. The compression along relevant dimensions $\mathrm{comp}r_{\mathrm{rel}}^{A},\mathrm{comp}r_{\mathrm{rel}}^{B}$ and irrelevant dimensions $\mathrm{comp}r_{\mathrm{irrel}}^{A},\mathrm{comp}r_{\mathrm{irrel}}^{B}$ as well as the context offset parameter context_offset are multiplied with the respective blocks in this feature matrix:

$$X=\left[\begin{array}{l} X_{A}\\ X_{B} \end{array}\right]=\left[\begin{array}{lll} c_{A} & \left(1-compr_{rel}^{A}\right)b_{A} & \left(1-compr_{irrel}^{A}\right)l_{A}\\ context\_offset*c_{B} & \left(1-compr_{irrel}^{B}\right)b_{B} & \left(1-compr_{rel}^{B}\right)l_{B} \end{array}\right]$$

The rotation parameter determines the extent to which the representation of the first task is rotated into the frame of reference of the second task:

$$R_{A}\left(\theta \right)=\left[\begin{array}{lll} 1 & 0 & 0\\ 0 & cos\left(\theta \right) & sin\left(\theta \right)\\ 0 & -sin\left(\theta \right) & cos\left(\theta \right) \end{array}\right]$$

This rotation was applied to $X_{A}$ in a subsequent step, so that the full model was given by:

$$X_{param}=\left[\begin{array}{lll} {}[c_{A} & \left(1-compr_{rel}^{A}\right)b_{A} & \left(1-compr_{irrel}^{A}\right)l_{A}]R_{\theta }\\ context\_offset*c_{B} & \left(1-compr_{irrel}^{B}\right)b_{B} & \left(1-compr_{rel}^{B}\right)l_{B} \end{array}\right]$$

We fit RDMs derived from this model to neural RDMs using a constrained optimization procedure (fmincon in MATLAB) with least-squares cost function. As the procedure is sensitive to the choice of starting values, we averaged over 1000 independent runs with random starting values. We then performed group-level inference on the distribution of best-fitting parameter values, where the overall compression index was defined as the log of the ratio between compression along the relevant and irrelevant dimensions:

$$compression=log\left(\frac{compr_{rel}}{compr_{irrel}}\right)$$

These were used to visualize the representational geometries of the best fitting RDMs via projection into three dimensions with classical Multi-Dimensional Scaling (MDS).

##### fMRI RSA: Embedding dimensionality

We performed Singular Value Decomposition (SVD) on the patterns of BOLD activity across voxels within each cross-validated ROI and calculated the cumulative explained variance based on the squared singular values to obtain an estimate of the embedding dimensionality (Jazayeri and Ostojic, 2021) of the neural activity patterns. To test whether the directions of largest variance were aligned with the task-diagnostic dimensions of context, branchiness and leafiness, we repeated the regression-based RSA within each cross-validated candidate region after successively removing components, starting with the smallest one. This truncated SVD allowed us to identify the minimal number of components required to successfully decode a factorised representation from the neural data.

##### fMRI RSA: Correlations between brain and behavior

We performed a correlation analysis (Kendall’s tau) to quantify the extent to which orthogonal representations at the neural level predicted accurate, axis-aligned behavioral responses. We analyzed human choice patterns by computing behavioral data RDMs from the probabilities of responding “plant” to trees in each condition, i.e., as a function of each stimulus’ distance to bound along the irrelevant and relevant dimension in each context. Building on previous work (Flesch et al., 2018) we fit two model RDMs to human choice patterns, called the *factorised* and *linear* models. In the factorised model, choices were aligned with the ground-truth boundaries, whereas in the linear model, a “diagonal” boundary was applied to both contexts, corresponding to the single linear boundary that optimized for accuracy while ignoring the context (yielding ∼70% correct). Fitting the factorised model to behavior yielded an “axis-alignment score,” indicating whether the participant’s decision boundaries were aligned with the ground truth. We tested at the group level whether the extent to which neural geometries could be explained by the orthogonal model (neural factorisation score) significantly covaried with the extent to which the factorised model explained human choices (axis alignment score).

##### fMRI RSA: Comparison of patterns across regions

To test statistically whether patterns differed between EVC and DLPFC/PPC/MCC, we performed random effects Bayesian model selection using the VBA toolbox for MATLAB (seek Key resource table). We created three regression models, consisting of (1) the branchiness and orthogonal RDM, (2) only the branchiness RDM and (3) only the orthogonal RDM. These were fit to the neural RDMs in EVC, DLPFC, PPC and MCC. We then approximated the log model evidences with the subject-specific negative BIC scores derived from the individual regression model fits. With these estimates, we performed random effects Bayesian model selection (RFX-BMS) to obtain exceedance probabilities – the probability that one model explains the data better than its competitors- and estimated model frequencies – the proportion of subjects explained by each model. We report protected exceedance probabilities, which correct the exceedance probabilities to reduce the possibility that an effect is observed due to chance.

##### fMRI MVPA: Decoding of relevant and irrelevant dimensions

We performed a decoding analysis to assess whether a classifier trained on the relevant dimension in one task could decode the same dimension in the other task where it was irrelevant. In theory, this should only be possible in EVC, which represents both feature dimensions irrespective of context, but not in our fronto-parietal areas of interest where irrelevant dimensions were (partially) suppressed. We first obtained single-trial estimates from a whole-brain GLM estimated on the neural data. We trained a linear support vector machine (SVM) with binary outputs at single subject level with leave-one-run-out cross validation on the t-maps obtained from this GLM. Within each run, patterns were first standardized and denoised by removing all but the first n principal components required to explain 95% of variance. Then, the classifier was trained to predict the choice-diagnostic label of the relevant dimension (for example, not leafy versus leafy) and its accuracy was assessed on data from the held-out run. We tested whether it could predict the relevant dimension of the task it had been trained on, the irrelevant dimension of the same task, and the relevant and irrelevant dimensions of the task it had not been trained on. Decoding accuracies were averaged within subjects across all held-out test sets and across tasks. To assess significance of the decoding performance, we performed group-level t test against a chance level of 0.5 across subjects.

#### Neural network simulations

##### Accuracy

The network was trained to predict the value of the relevant feature dimension in each context, defined as the signed distance to a category boundary, y∈[−2,−1,0,1,2]. In contrast, human participants had to accept/reject stimuli based on this signed distance. For comparison between neural networks and human participants, we quantified the network’s accuracy as the match between the signs of the network’s predictions and the ground truth:

$$Accuracy∶=\frac{1}{N}\sum_{i=1}^{n}1_{\left(\left({\hat{y}}_{i}>0\right)==\left(y_{i}>0\right)\right)}$$

##### Endpoint weight norm and relative weight change

Every 100 epochs during training, we computed the Frobenius norm of the hidden layer weights

$$W_{hidden\;F}=\sqrt{\sum_{i=1}^{m}\sum_{j=1}^{n}{\left|w_{ij}\right|}^{2}}$$

and their relative change with respect to the norm at initialisation. This allowed us to assess whether the network operated in the rich or lazy regime, corresponding to low and high norm solutions. The weight change relative to initialisation was quantified by computing how the norm of the hidden layer weights changed from random initialisation to the endpoint of training.

$$log\left(\Delta W_{hidden\;F}\right)=log\left(\sqrt{\sum_{i=1}^{m}\sum_{j=1}^{n}{\left|w_{ij}^{t=T}-w_{ij}^{t=0}\right|}^{2}}\right)$$

##### Neural network representational similarity analysis

We performed RSA on the hidden layer activity patterns to assess how training sculpted the representations formed by the neural network. For each individual run, we calculated RDMs based on the hidden layer activity patterns evoked by inputs covering all combinations of feature values and contexts. The resulting 50x50 RDMs captured the Euclidean distances between all possible pairs of stimuli in the high-dimensional space spanned by the hidden units (after the ReLU nonlinearity). We visualized these geometries by projecting the group-level RDM, averaged across independent runs, down into three dimensions using metric MDS.

##### Neural network RSA: Quantifying hidden layer geometries

To quantify the extent to which hidden layer geometries exhibited patterns consistent with our hypotheses, we performed a linear regression of the hidden layer RDMs onto a set of model RDMs. There were three model RDMs in total, (1) a grid model, encoding the stimulus spaces as two parallel grids, separated by the context, (2) an orthogonal model, encoding the task relevant dimensions as two orthogonal 1D manifolds and (3) a parallel model, encoding the same information as the orthogonal model, but rotated into the frame of reference of the response (i.e., a “magnitude” representation). These were identical to the grid, orthogonal and parallel models described above in the section Representational similarity analysis of human fMRI. The lower triangular form of these models was z-scored and entered into a linear multiple regression model to predict the lower triangular form of the hidden layer RDM:

$$RDM_{brain}=\beta_{0}+\beta_{1}\;RDM_{grid}+\beta_{2}\;RDM_{orth}+\beta_{3}\;RDM_{parallel}$$

This procedure was repeated for each individual training run, yielding a distribution of regression coefficients that permitted statistical inference on the relative difference between predictors as well as their difference from zero. We tested whether two models differed in their extent to which they covaried with the hidden layer RDM by performing Wilcoxon Signed Rank tests on their corresponding beta estimates. A nonparametric test was chosen due to the observed violation of the normality assumption. We applied this analysis to models with different initial weight scale, enabling us to investigate the impact of the training regime (rich or lazy) on the emerging representations. For a more nuanced analysis of the hidden layer patterns, we fit the same parameterised model described above for the fMRI data to the hidden layer activity.

##### Neural networks: Embedding dimensionality of hidden layer activity patterns

We used SVD to investigate the embedding dimensionality of the hidden layer activity patterns (Jazayeri and Ostojic, 2021). SVD was applied to the stimulus-by-unit matrix of hidden layer responses to all combinations of feature values and context. We visualized the cumulative variance explained based on the squared singular values (i.e., the eigenvalues of the response matrix) as Scree plot and performed the Elbow method to obtain a qualitative estimate of the embedding dimensionality. Next, we performed truncated SVD to assess the task-diagnosticity of the first k directions of variation in the response matrix. For this, we reconstructed the hidden layer response matrix, keeping only the first k singular values with k ranging from 1 to 27 (i.e., the number of input units). We then generated new outputs from the network by passing this lower-dimensional activity pattern on to the output unit. Lastly, we calculated the accuracy as the mismatch between these outputs and the ground truth, using the formula described in the section above. This allowed us to assess, separately for the rich and lazy regime, the extent to which removing components from the hidden layer responses reduced the network’s performance. The hypothesis was that more components would be needed in the lazy compared to the rich regime to maintain equal task accuracy.

##### Neural network hidden unit selectivity and axis alignment

To investigate task selectivity of hidden layer units, we capitalised on the property of ReLU nonlinearities that they map negative inputs to zero. We defined task-selectivity for the neural network as a non-zero response to stimuli in one context and zero response to all stimuli in the other context. Stimulus selectivity irrespective of context was defined as having a non-zero response in both contexts. We calculated these sensitivity indices at initialisation and after training to ensure that the initialisation scheme did not pre-partition the hidden layer in the absence of a training objective. Dead units were defined as returning zero for all stimuli (all combinations of feature values and context). From this, we calculated the proportion of units that were either dead, task- or stimulus-selective. To visualize response profiles, we averaged activity within these sub-populations, constructed a response matrix of these averages separately for each context (with rows corresponding to y location, columns to x-locations of stimuli and the value corresponding to the average activity of a sub-population) and plotted the group level average (mean across independent runs) as heatmaps. For this, we focused on the two most extreme weight initialisations, 0.01 and 3, corresponding to learning in the rich and lazy regime, respectively. Lastly, to quantify the extent to which these response patterns were axis aligned (i.e., whether units responded to relevant but not irrelevant dimensions), we concatenated the two vectorised task response matrices, constructed RDMs based on pairwise differences in magnitude and regressed them against two model RDMs, (1) the factorised and (2) linear models. In the factorised model, unit responses scaled with context-dependent relevant dimensions (i.e., with x-location in context A and y-location in context B). In the linear model, activity scaled jointly with both dimensions irrespective of context. We fitted the model at the level of individual runs. To assess which model RDM covaried stronger with the observed neural responses, we performed a Wilcoxon Signed Rank test on the difference between beta estimates for the factorised and linear model. To assess whether this difference was dependent on the initialisation scheme, we performed the same test on the difference of differences.

##### Neural network context weight correlations

Our theory predicted that the network could learn the gating scheme via anti-correlated context weights. To test this empirically, we calculated the Pearson correlation between task A and task B weights from the input to the hidden layer at the level of single runs both at initialisation and after the last training epoch. We repeated this analysis on the sub-populations of task-specific and task-agnostic units, expecting weights into the former to be stronger anti-correlated. We visualized the distribution of single-run correlation coefficients together with a Kernel-Density-Estimate computed with the kdensity function from the Seaborn package.

##### Neural network ablation study

We performed an ablation study to investigate how critical task-sensitive and stimulus-sensitive units were for multi-task performance. More specifically, for each collected run, we set either the sub-population of task-specific or task-agnostic units to zero, performed a forward pass through the ablated network and computed its loss and accuracy.

#### Non-human primate data

##### Representational similarity analysis of NHP electrode recordings

We created pseudo-populations by concatenating all recorded units, separately for monkey A and monkey F. Unit-by-stimulus response matrices were obtained by averaging activity across trials for each stimulus type (6 motion directions ^∗^ 6 colors ^∗^ 2 contexts = 72 entries). RDMs were constructed from these matrices using the Euclidean distance measure. For all reported analyses, we focus on activity averaged over the second half of the trial (300-600ms) where task factorisation was strongest, an observation consistent with previous reports of dynamic encoding of different task variables throughout a trial (Aoi et al., 2019). We fitted the same set of candidate model RDMs to this dataset as previously to RDMs obtained from human fMRI data (see above). For statistical inference, we created a null distribution by randomly permuting the trial labels and repeating this regression-based RSA 1000 times. We calculated p values from the proportion of permutations that yielded regression coefficients larger than the one observed on the original data.

##### Individual unit selectivity and axis alignment of NHP electrode recordings

We assessed task selectivity of individual units using a standard regression-based approach. Mean activity of each unit was regressed against four predictors, coding for color and motion direction separately for each context:

$$y_{unit}=\beta_{0}+\beta_{1}\;colour_{colour\;task}+\beta_{2}\;motion_{colour\;task}+\beta_{3}\;colour_{motion\;task}+\beta_{4}\;motion_{motion\;task}$$

Selectivity was defined as having a significant regression coefficient for the variable of interest. Due to the substantial number of tests, we performed FDR correction to correct for multiple comparisons. We distinguish between diverse types of selectivity. Task-selectivity was defined as having a significant regression weight only for the relevant feature dimension (i.e., only for motion in the motion task or color in the color task). Task-agnosticity was defined as having significant coefficients for both dimensions. Furthermore, we identified units that were selective only to color or motion, irrespective of context, and defined non-specific selectivity as having significant regression weights that do not fall into any of the above categories. As for the hidden units in the neural network, we again plotted the different proportions of selectivity patterns of units within a pseudo population and visualized the response profile of task and stimulus selective units by averaging the activity within a sub-population separately for each combination of feature values (color, motion) and context. Axis alignment of these response matrices was assessed by regressing them against the factorised and diagonal model as previously described for the neural network (see above). We assessed the embedding dimensionality of the patterns observed in monkey FEF using the same truncated SVD approach described above for the human fMRI data.

##### Decoding of relevant and irrelevant dimensions

To assess whether task-irrelevant dimensions were filtered out in the NHP data, we performed a decoding analysis that was similar to the one described above for the fMRI data. We first divided the trials of each unit into a first and second half. As data from the units in the original dataset had been recorded during different sessions, we first created fully counterbalanced pseudo trials. We generated 1440 pseudo trials by sampling each condition from the set of recording units and creating vectors of condition-by-unit activity that represented individual trials as if activity from these units had been recorded simultaneously. We repeated the procedure for the second half of the dataset, thus yielding 1440 training and 1440 test trials. The data was standardized and denoised by removing all but the first n principal components required to explain 90% of the variance. We then trained a linear SVM on the relevant dimension of the NHP dataset with two-fold cross-validation and assessed its decoding performance on the relevant and irrelevant dimensions in the held-out dataset. Statistical significance was assessed with a permutation test in which we computed test performance after randomly shuffling the labels (1000 permutations). Chance was defined as the average performance on these shuffled datasets (roughly 0.5%) and p values were computed from the proportion of trials in which the decoding accuracy exceeded the one observed on the original data.

## Data Availability

Code to replicate human experiments, simulations, and analyses is available on Github (https://doi.org/10.5281/zenodo.5807610). Raw NHP data is freely available under https://www.ini.uzh.ch/en/research/groups/mante/data.html. In line with local ethics guidelines, preprocessed and anonymized group-level human fMRI data is available on OSF: https://www.doi.org/10.17605/OSF.IO/7VCGH

## Supplemental information

Document S1. Figures S1–S6 and Table S1

Document S2. Article plus supplemental information
