# Orthogonal representations for robust context-dependent task performance in brains and neural networks

**DOI:** 10.1016/j.neuron.2022.12.004

**Published:** 2022-12-21

**Authors:** Timo Flesch, Keno Juechems, Tsvetomira Dumbalska, Andrew Saxe, Christopher Summerfield

## Main text

(Neuron *110*, 1258–1270.e1–e11; April 6, 2022)

We thank the authors of Mante et al. (2013) for alerting us to a labeling error in the presentation of our reanalysis of their data. Unfortunately, we switched the labels for "colour" and "motion" in Figures 4 and 6 as well as Figures S4 and S5, which provoked a discrepancy with the findings presented in the original report. The figures have now been corrected online. We apologize for any confusion this may have caused.

**Figure:**
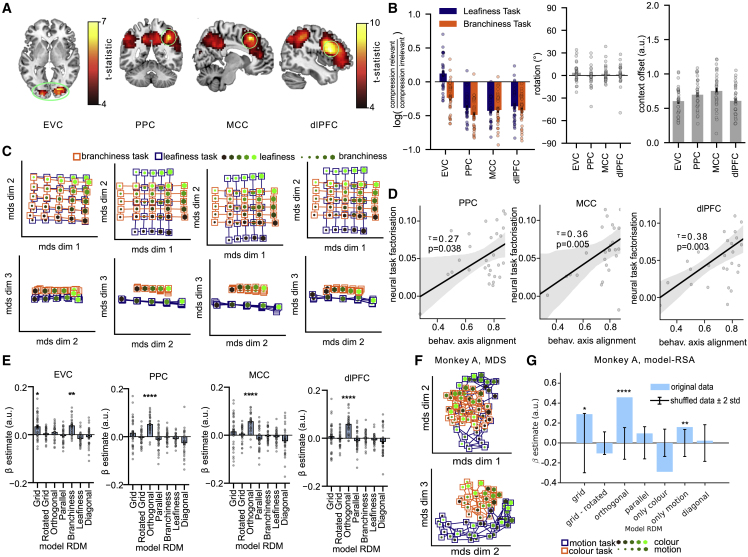
Figure 4. Task representations in human fMRI and macaque unit recordings (corrected)

**Figure:**
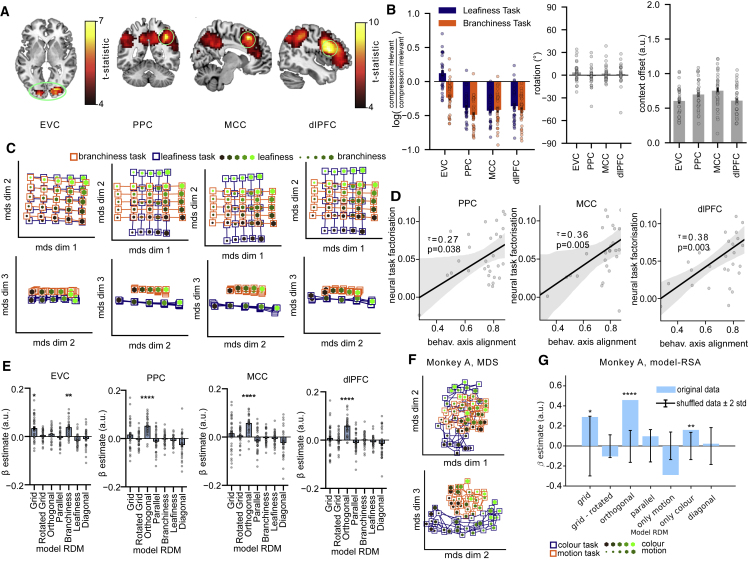
Figure 4. Task representations in human fMRI and macaque unit recordings (original)

**Figure:**
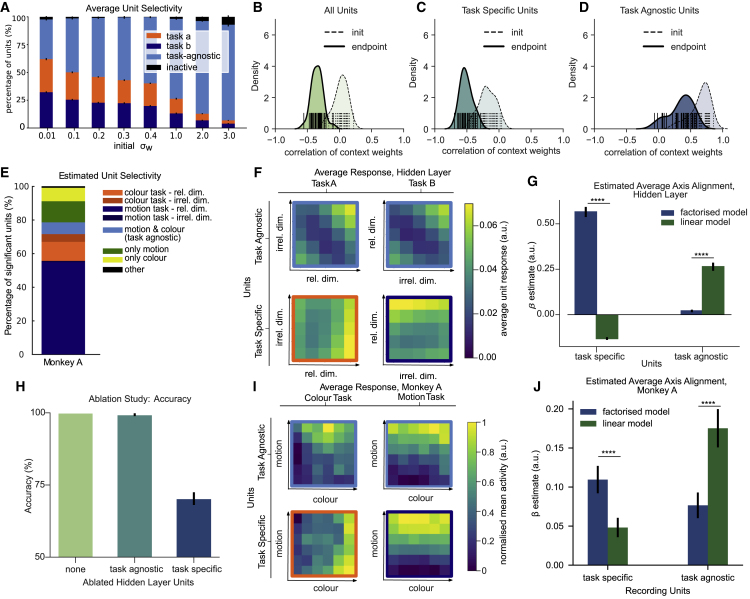
Figure 6. Neural network and NHP data in support of gating theory (corrected)

**Figure:**
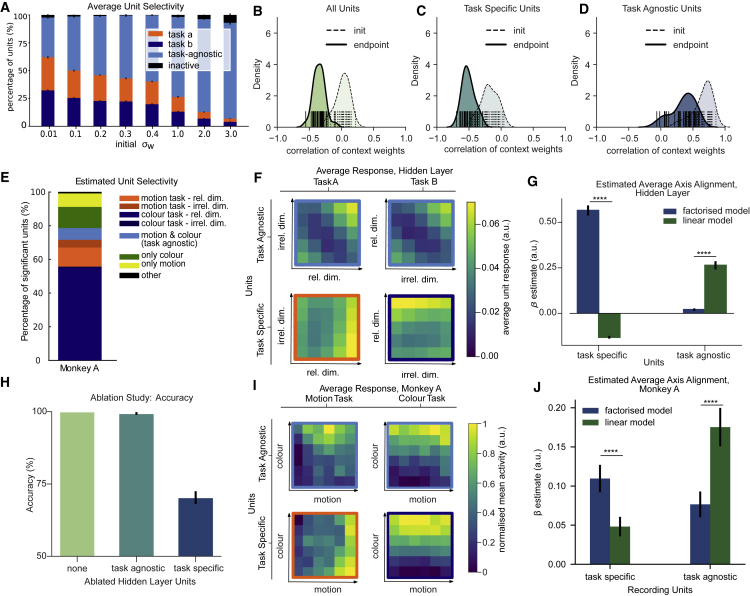
Figure 6. Neural network and NHP data in support of gating theory (original)

**Figure:**
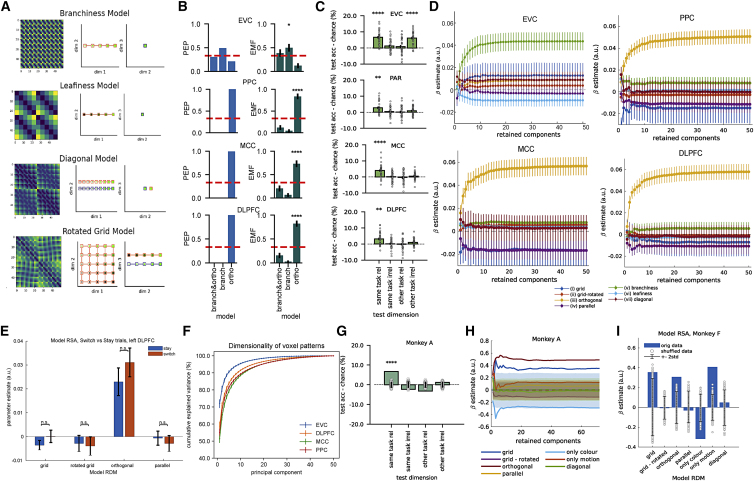
Figure S4. Control analyses on the human fMRI and NHP datasets (corrected)

**Figure:**
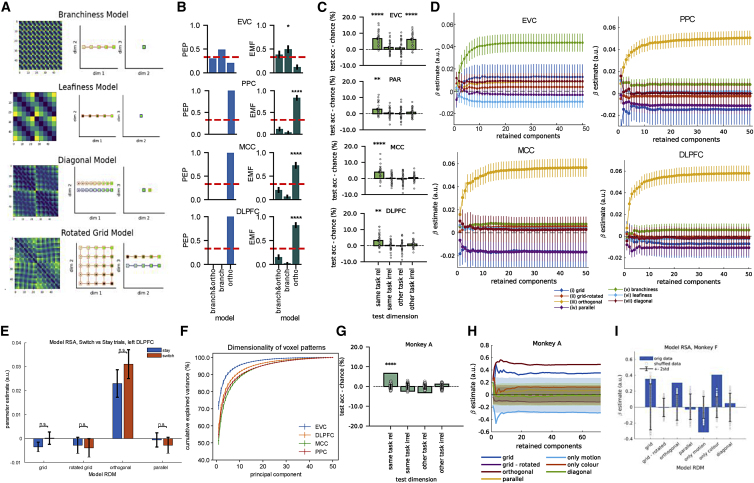
Figure S4. Control analyses on the human fMRI and NHP datasets (original)

**Figure:**
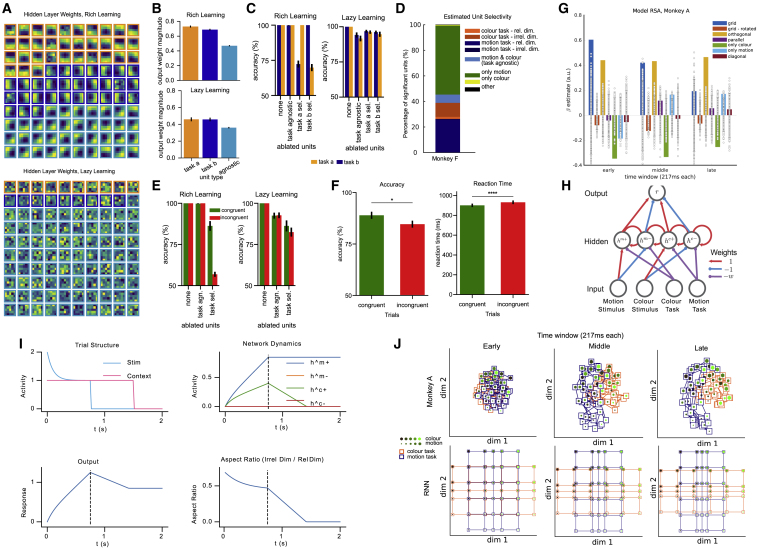
Figure S5. Gating in MLPs, NHPs and RNN model (corrected)

**Figure:**
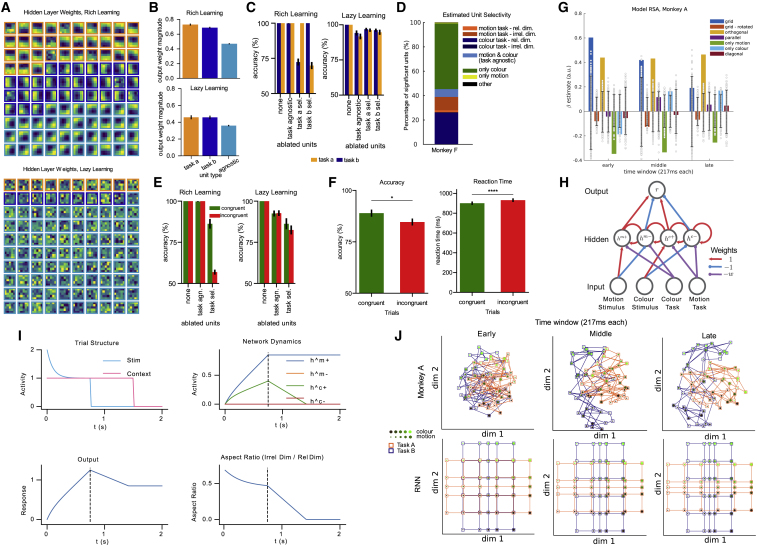
Figure S5. Gating in MLPs, NHPs and RNN model (original)

