# Supplementary material for: Orthogonal representations for robust context-dependent task performance in brains and neural networks
Source: Neuron. 2022 Apr 6;110(7):1258–1270.e11. doi: 10.1016/j.neuron.2022.01.005 (PMC8992799; doi:10.1016/j.neuron.2022.01.005)
Supplement: Document S1. Figures S1–S6 and Table S1 [file mmc1.pdf]

Neuron, Volume *110*

Supplemental information

Orthogonal representations for robust context-dependent task performance in brains and neural networks

Timo Flesch, Keno Juechems, Tsvetomira Dumbalska, Andrew Saxe, and Christopher Summerfield

Supplementary Information for

Orthogonal representations for robust context-dependent task performance in brains and neural networks

Timo Flesch, Keno Juechems, Tsvetomira Dumbalska, Andrew Saxe, Christopher Summerfield

Correspondence to:
timo.flesch@psy.ox.ac.uk, a.saxe@ucl.ac.uk, christopher.summerfield@psy.ox.ac.uk

This PDF file includes:

Figs. S1 to S6
Table T1

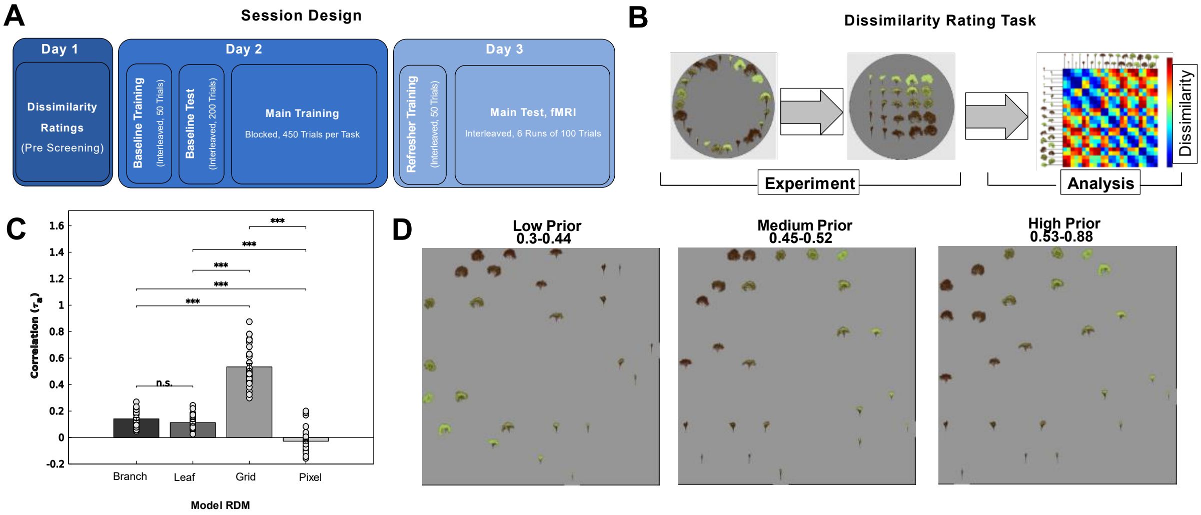

Fig. S1. Experimental design and grid prior analysis. (related to Fig. 1). (**A**) Session Design. Participants completed three sessions carried out over consecutive days. All participants underwent a screening task (day1) in which they were asked to perform dissimilarity ratings on tree stimuli. Those who showed strong evidence for being aware of the dimensions of branchiness and leafiness (assessed by a "grid score", see next figure) were invited to the remaining parts of the study. On day 2, participants received a lengthy blocked training curriculum, preceded by a brief familiarisation phase and evaluation (baseline training and test) to measure the effectiveness of the training phase. On day 3, participants received a brief refresher training, before they underwent fMRI scanning during which they completed six interleaved blocks of test trials. See methods sections for additional details. (**B**) Dissimilarity Rating Task & RSA. Participants were asked to arrange tree stimuli via mouse drag & drop in a circular arena such that distances between trees corresponded to how dissimilar they were perceived (left and middle panel). From these ratings, we constructed RDMs at single subject level. These RDMs were correlated with model RDMs assuming that participants were (i) only aware of branchiness, (ii) only aware of leafiness, (iii) aware of the full 5x5 grid of branchiness and leafiness or (iv) made judgements based on pixel similarity. We describe the extent to which the third model explains the data as "grid score". In Flesch et al, 2018, we reported interactions between training effectiveness and grid score. We thus only invited participants with a grid score higher than the median grid score (tau=0.18) from the previous study. All screened participants exceeded this threshold. (**C**) Correlation coefficients between subject ratings and model RDMs. The grid model explained the data best, indicating that participants were on average aware of the data-generating dimensions. (**D**) MDS on dissimilarity ratings, divided into participants with low, medium and high grid score. All groups showed evidence for awareness of the dimensions branchiness & leafiness, and their grid-like relationship with each other.

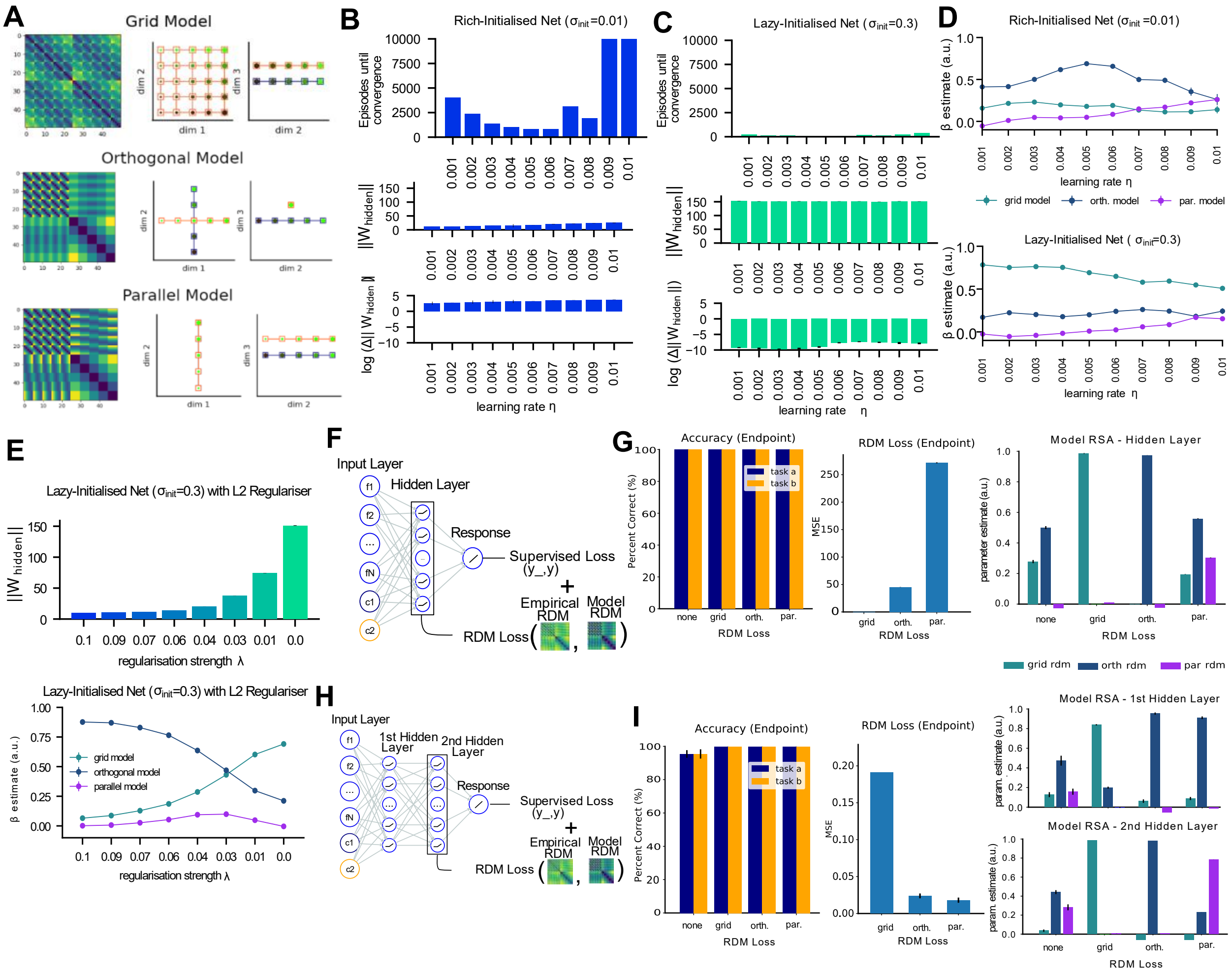

Fig. S2. Additional MLP simulations and controls (Related to Fig. 2). (**A**) Model RDMs used for Representational Similarity Analysis. The grid model encoded both feature dimensions and context along three dimensions. The orthogonal model encoded only the context and the relevant feature dimensions. The parallel model was obtained by rotating one of the task representations from the orthogonal model by 90 degrees, corresponding to a shared encoding of the task/readout axis. (**B**) Time until convergence (top) and weight change (middle/bottom) as a function of the learning rate for a network initialized in the rich regime. (**C**) Same as (b) but initialized in lazy regime. Note that irrespective of learning rate, all nets converged faster than under (b) and had smaller weight changes. (**D**) RSA on hidden layer patterns as function of learning rate, for rich (top) and lazy (bottom) initialized nets, showing again that learning rate was not critical. (**E**) Lazy-initialised network with L2 regulariser, demonstrating that weight norm (top) and task-specificity of representations (bottom) can be controlled by a regulariser. (**F**) We equipped the network with an auxiliary objective ("RDM loss") which minimised the difference between patterns in the hidden layer and a candidate model RDM that encoded either grid-like, orthogonal or parallel representational schemes. (**G**) (left) Accuracy after convergence on the supervised objective, depending on chosen constrain on RDM. All models converged . (middle) Endpoint RDM loss after convergence on the supervised objective. All networks except for the one with parallel model RDM loss converged. (right) Model RSA. The models with grid and orthogonal schemes as target for the RDM loss learned the desired representations. The model trained with a parallel RDM as target in the RDM loss converged to orthogonal representations. (**H)** Same as (g) but for model with two hidden layers. This time, parallel representations could be enforced in the second layer, leading to orthogonal representations in the first layer.

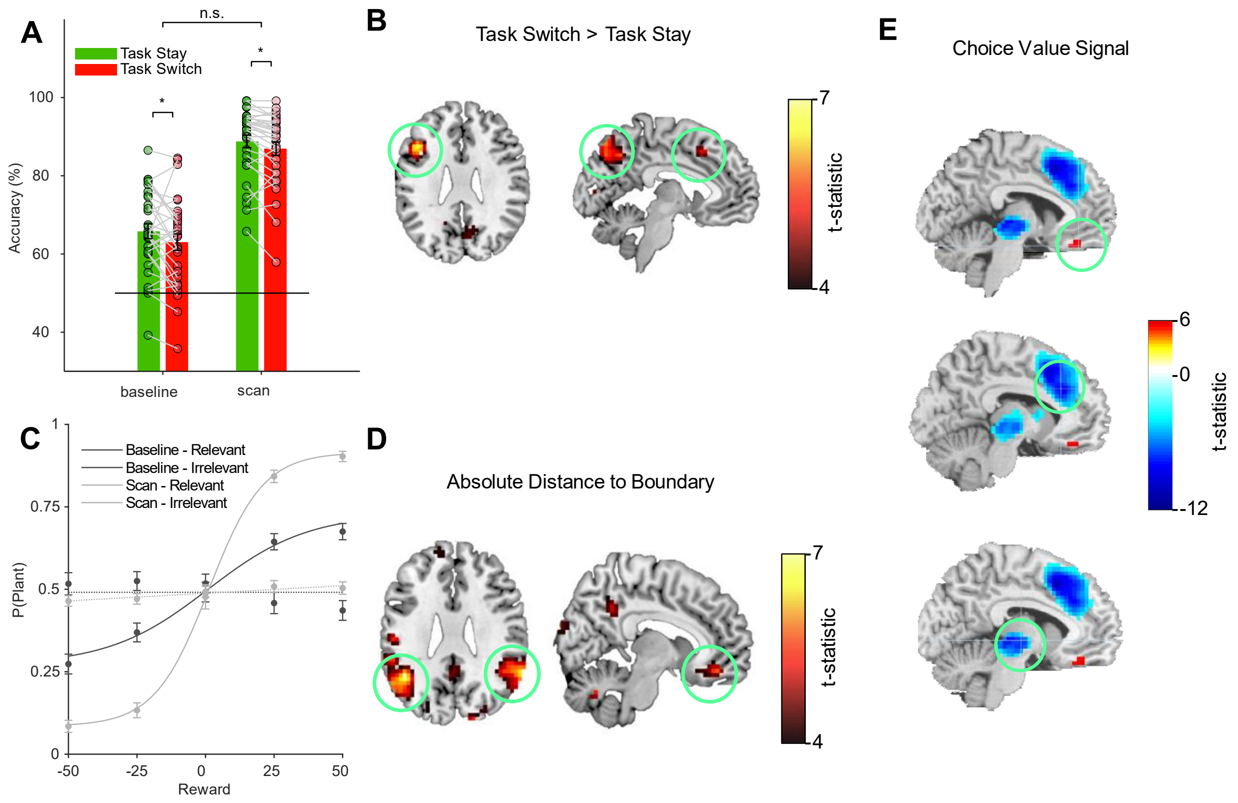

Fig. S3. Replication of standard univariate findings (Related to Fig. 4). (**A**) Behavioural switch cost. Participants were slightly worse on switch than stay trials at test, both during the baseline and later scanning session (*Accuracy Baseline, Switch < Stay: T(29)=2.057, p=0.048, d=0.266; Accuracy Scan, Switch < Stay: T(29)=2.715, p=0.011, d=0.211; Interaction Phase x Switch cost: T(29)=-0.668, p=0.509, d=-0.251*). (**B**) Univariate markers of switch cost. A whole-brain univariate contrast of switch vs stay trials revealed lusters in task-positive regions where activity was higher on switch than on stay trials. More specifically, we found significant clusters in Parietal Cortex *(BA7 : t(30) = 5.65, p < 0.001 (FWE corrected), cluster extent (kE) = 570, MNI coords = [-6, -74, 52]),* Supplementary Motor *Area (SMA t(30) = 5.03, p < 0.05, kE =66, [-6, 18, 46]))* and left Medial Frontal Gyrus *(MFG t(30)=6.55, p<0.01, kE = 124, [-44, 21, 28]))* (**C**) Behavioural sensitivity to relevant and irrelevant dimensions. Fitting logistic functions to the choice patterns along both dimensions revealed that, compared to the baseline, participants became much more sensitive to the task-relevant dimension after they had engaged in the blocked training phase *(Slope Relevant, Baseline: Z = 4.72, p = < 0.001, d = 0.873; Scan > Baseline: Z = 4.762, p = < Scan: Z = 2.705, p = 0.007, d = 0.494).* Participants were, however, much more sensitive to the relevant than irrelevant dimension at *test (Scan, Relevant > Irrelevant: Z = 4.782, p < 0.001, d = 0.873)*, and this sensitivity was higher compared to baseline *(Dimension x Phase Interaction: Z = 4.741, p < 0.001, d = 0.866)* (**D**) Univariate markers of absolute distance to category boundary. A GLM with parametric regressors for the absolute distance to category boundary (methods) revealed significant relationships between activity and distance to bound along the relevant, but not irrelevant feature dimensions. More specifically, we found significant clusters in bilateral Angular Gyrus *(left: t(30) = 6.79, p < 0.001, kE = 364, [60, -49, 28])* and the right Orbitofrontal Corex (t(30) = 5.46, p < 0.01, kE = 73, [8, 42, -14]), and to a lesser extent also in bilateral EVC *(left: t(30) = 5.15, p < 0.01, kE = 70, [-13, -98, 14]; right: t(30) = 6.55, p < 0.01, kE = 61, [18, -94, 21]*) as well as the Posterior Cingulate cortex *(t(30) = 5.05, p < 0.001, kE = 192, [4, -49, 35]).* (**E**) Univariate markers of choice value. A GLM with regressors for the choice and value of the stimuli revealed significant relationships between the interaction of choice and value and BOLD. Consistent with previous studies, we found clusters in ACC *(t(29) = -9.52, p < 0.001 uncorr , kE = 803 , [8,28,31]),* VMPFC *(t(29) = 4.33, p < 0.001 uncorr, kE = 23, [4.5,38.5,-21])* and the striatum *(t(29) = -8.88, p < 0.001, kE = 302 , [-2.5,-14,-3.5]).*

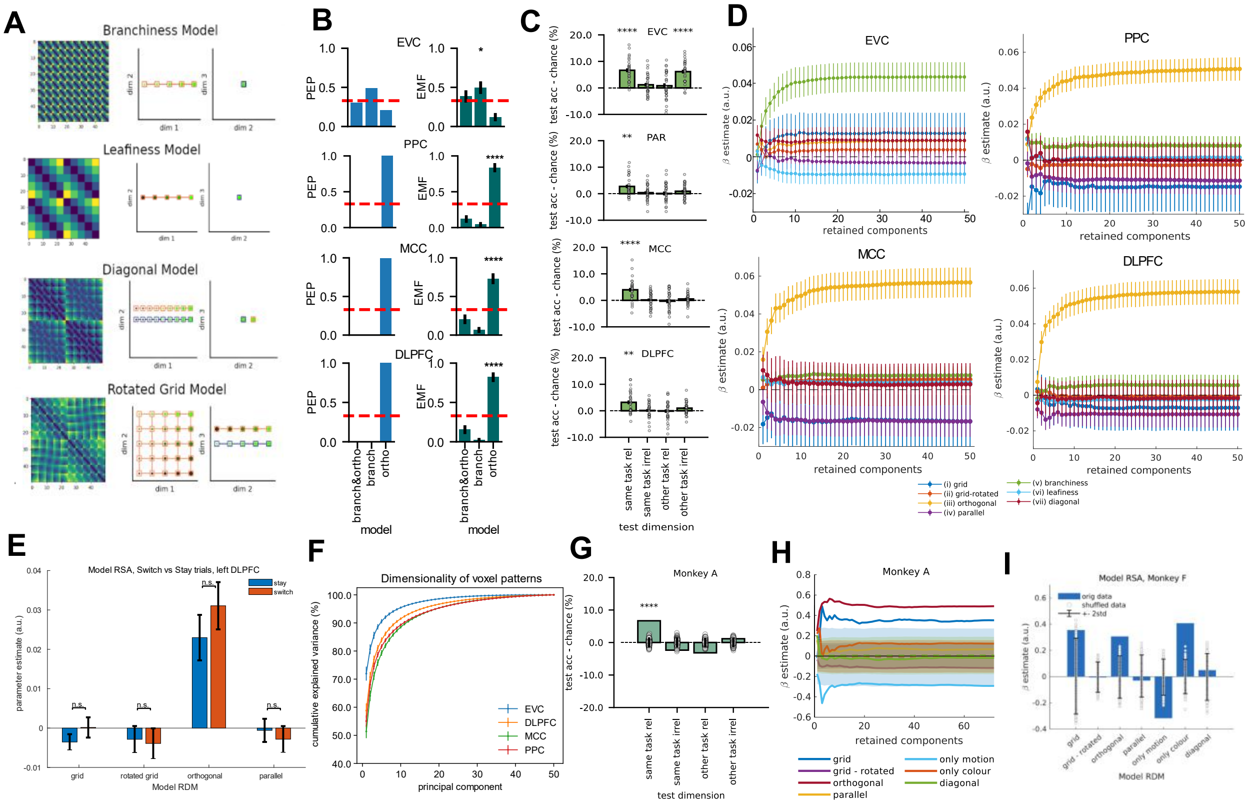

Fig. S4. Control analyses on the human fMRI and NHP datasets (Related to Fig. 4). (**A**) Control RDMs used in addition to the grid, orthogonal and parallel model (methods). (**B**) Protected exceedance probabilities (left) and estimated model frequencies (right) of a Bayesian model comparison between rich and lazy RDMs for the four candidate regions. Protected exceedance probabilities of comparisons between regions (not shown) implied that it was very unlikely that the same model explained patterns in EVC and DLPFC/PPC/MCC *(EVC & DLPFC: pep=3.29e-4, EVC & MCC: pep=0.0029, EVC & PPC: pep=1.91e-4)*. RFX BMS within each region confirmed again that the branchiness model explained most of the patterns in EVC, in contrast to the orthogonal model in DLPFC/PPC/MCC. Full statistical results are reported in Table T1. (**C**) Cross-validated decoding accuracies for a linear SVM trained on the relevant dimension on one task and evaluated on the relevant/irrelevant dimension of the same and the other task. Only in EVC, the same dimension can be decoded from the other task (where it was irrelevant), showing that the other regions suppressed task-irrelevant dimensions. Asterisks indicate p-values after Bonferroni-correction. (**D**) truncated SVD. (**E**) The univariate contrast of switch vs stay trials revealed a significant difference in BOLD in left DLPFC, an area where we had also observed evidence for factorised representations using the searchlight RSA approach. We therefore tested whether the extent to which task representations were factorised (i.e. lied on orthogonal manifolds) differed between switch and stay trials. The difference, however, was not significant. (**F**) Pattern dimensionality in the four ROIs. (**G**) Same as (C) but on pseudo-trials generated from Monkey A data(methods) again showing that only task-relevant dimension was represented in the neural pattern. (**H**) Same as (D) but for Monkey A. (**I**) RSA for Monkey F, showing that in contrast to Monkey A (main text), patterns encoded predominantly colour irrespective of context.

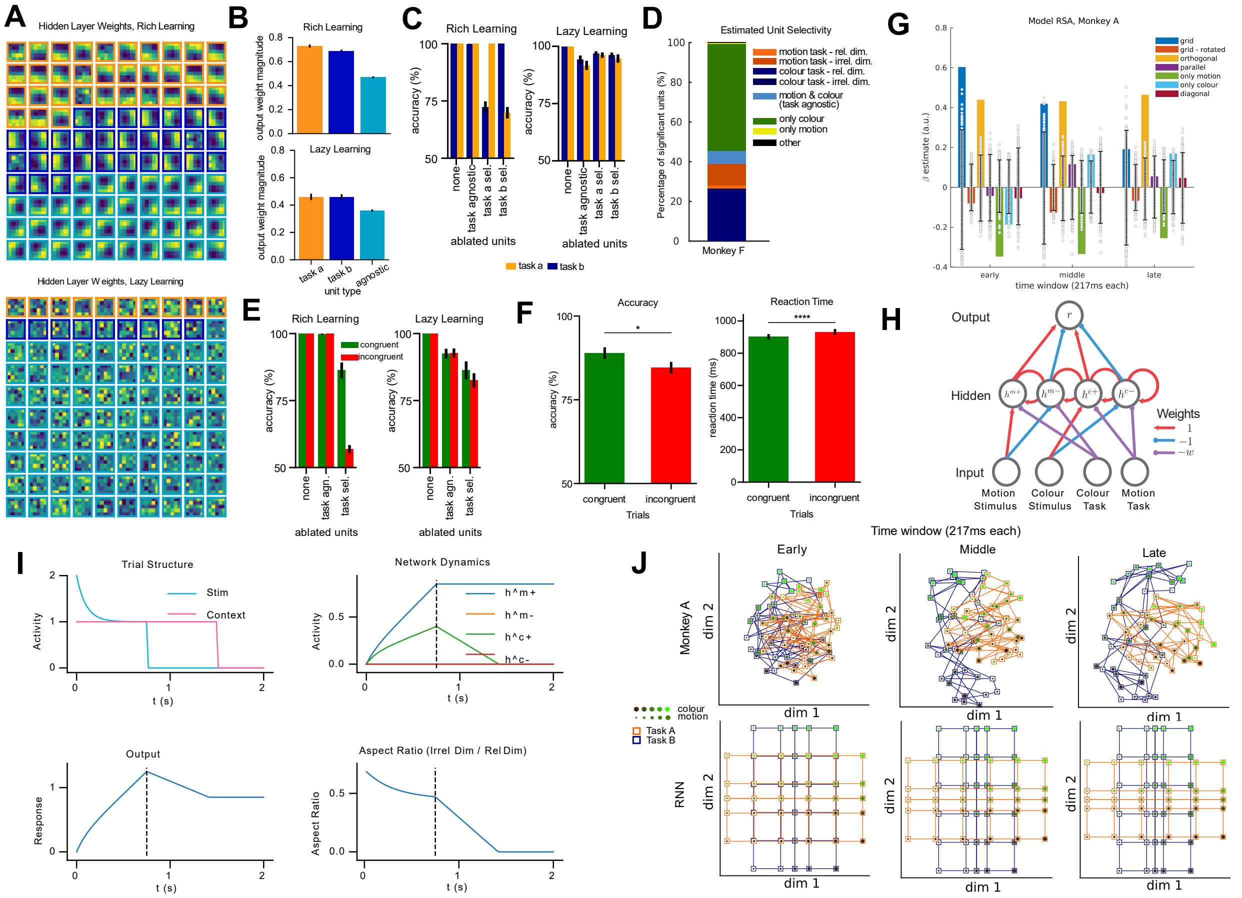

Fig. S5. Gating in MLPs, NHPs and RNN model (Related to Fig. 6). (**A**) Input-to-hidden weights, reshaped to resemble the dimensionality of the inputs and divided into units that are active only in task A (orange), task B (dark blue), or both (light blue frame). Note that task-specific units have axis-aligned receptive fields under rich (top) but not under lazy learning (bottom), where selectivity his highly heterogenous. (**B**) Magnitude of task-specific and task-agnostic weights under rich (top) and lazy (bottom) learning, showing that the network relies more on task-specific units, especially under rich learning. (**C**) Ablation study, with performance shown separately for task A and B. Under rich learning, where task-specific units are axis aligned, removing them only affects performance on the task they are selective for. (**D**) Distribution of unit selectivity for monkey F; most recorded units are colour-selective. (**E**) Ablation study with performance separately on congruent (same response in both contexts) and incongruent (different responses in task A and B) trials. Under rich learning, task-specific units are axis aligned, and task-agnostic units encode congruent trials. Hence, when task-specific units are removed, the network still performs well on congruent, but not incongruent trials. (**F**) Congruency effect in human accuracy and RT. Participants were better (T(30) = 2.68, p = 0.012) and faster (T(30) = -5.11, p<0.0001) on congruent trials**.** (**G**) Model RSA on NHP data, separately for early, middle and late time windows within the stimulus interval, suggesting that the neural code transforms from a grid-like to an orthogonal and task-specific representation. (**H**) RNN version of our neural network model. (**I**) RNN dynamics throughout a simulated trial. Top left: Stimulus and context are presented for 750ms, followed by delay (1s) where only context is present. Top right: Hidden layer dynamics during motion task trial. We observe a gradual integration of motion information in the motion-sensitive unit, and, to a lesser extent, colour information in the colour-sensitive unit. After stimulus offset (dashed line), the irrelevant dimension (colour) is gradually suppressed by the context signal. Bottom left: Gradual integration of a category signal in the output unit, which remains roughly constant after stimulus offset. Bottom right: Aspect ratio between activity encoding the irrelevant and relevant dimensions respectively, indexing the amount of compression along irrelevant dimensions. The aspect ratio decreases during the stimulus interval as irrelevant and relevant feature information are integrated at different rates (top right plot). It decreases more rapidly after stimulus offset (dashed line) as the context signal filters out any task-irrelevant information that is still present. (**D)** MDS on monkey and RNN RDMs averaged over early, middle and late time windows within the stimulus interval.

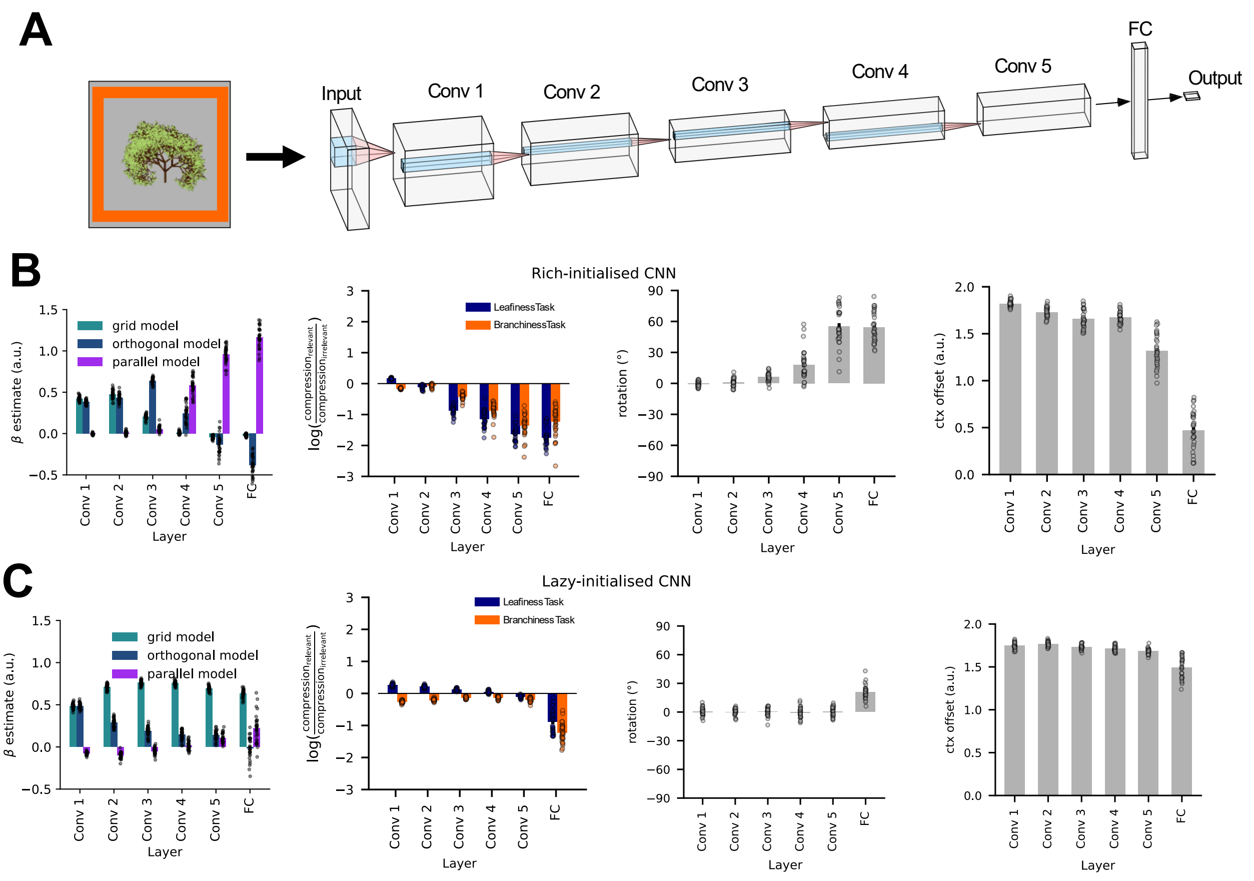

Fig. S6. Convolutional Neural Network (CNN) trained on the same stimuli as human participants (Related to Fig. 3). (**A**) Network architecture. We trained a feedforward CNN with five convolutional and one full-connected layer on the trees task. The network received RGB images of trees surrounded by an orange/blue frame to signal context, and had to predict the task-relevant label (level of branchiness/leafiness). The network was trained either in the rich (small initial weights) or lazy (large initial weights) regime. (**B**) RSA results for network trained in rich regime. Left: Coefficients of grid, orthogonal and parallel model obtained from Linear Regression performed on patterns from each layer. Each dot corresponds to a single trained neural network (30 in total). Early layers encode both feature dimensions, followed by orthogonal representations in intermediate layers and parallel representations closer to the readout. The conversion from task-agnostic representations of the inputs into task-specific representations of the rules was confirmed by fitting the compression, rotation and offset parameters of the fully-parameterised model RDM (middle and right plots).
(**C**) Same as (B), but for model trained in the lazy regime. All convolutional layers had task-agnostic representations, while the FC layer showed signs of task-specific representations.

Table T1. Results of Bayesian Model Comparison between brain regions (Related to Fig. 4, see STAR methods for details).

**ROI**		**Protected Exceedance Probability**			**Estimated Model Frequencies**	
	Branchiness & Orthogonal	Branchiness	Orthogonal	Branchiness & Orthogonal	Branchiness	Orthogonal
EVC	0.302	**0.489**	0.209	0.38±0.08, z=0.8, p=0.21	**0.5±0.08 z=2.29, p=0.01**	0.12±0.05 z=-3.08, p=0.999
DLPFC	0.0	0.0	**1.0**	0.15±0.06, z=-2.86, p=0.99	0.03±0.02, z=-4.28, p=1	**0.82±0.06, z=4.51, p<0.0001**
MCC	0.003	0.002	**0.995**	0.20±0.06, z=-2.00, p=0.97	0.07±0.04, z=-3.69, p=1	**0.73±0.07, z=4.19, p<0.0001**
PPC	0.0	0.0	**1.0**	0.12±0.05, z=-3.08, p=0.99	0.04±0.03, z=-4.24, p=1	**0.84±0.06, z=4.57, p<0.0001**
